# Comparison of LC-MS methods for the quantitation of ciguatoxins in fish – A collaborative study

**DOI:** 10.1016/j.fochx.2025.103277

**Published:** 2025-11-09

**Authors:** Astrid Spielmeyer, Vincent Hort, J. Sam Murray, Cintia Flores, Andres Sanchez-Henao, Emillie M.F. Passfield, Caroline Desbourdes, Lourdes Barreiro-Crespo, Mònica Campàs, Jorge Diogène, Fernando Real Valcárcel, Jean Turquet, Christopher R. Loeffler, Maria Rambla-Alegre

**Affiliations:** a German Federal Institute for Risk Assessment, Reference Centre for Food and Feed Analysis, National Reference Laboratory for the Monitoring of Marine Biotoxins, Max-Dohrn-Str. 8-10, 10589 Berlin, Germany; b French Agency for Food, Environmental and Occupational Health and Safety (ANSES), Laboratory for Food Safety, Pesticides and Marine Biotoxins Unit, 14 rue Pierre et Marie Curie, 94701 Maisons-Alfort, France; c Cawthron Institute, Private Bag 2, Nelson 7040, New Zealand; d Institute of Environmental Assessment and Water Research (IDAEA-CSIC), Mass Spectrometry Laboratory/Organic Pollutants, Jordi Girona 18, 08034 Barcelona, Spain; e IRTA, Marine and Continental Waters, Ctra. Poble Nou, km. 5.5, 43540 La Ràpita, Spain; f University Institute of Animal Health and Food Safety (IUSA), University of Las Palmas de Gran Canaria, Ctra. Transmontaña S/N, 35413 Arucas, Spain; g Universitat Rovira i Virgili, Department of Analytical Chemistry and Organic Chemistry, C/Marcel·lí Domingo S/N, 43007 Tarragona, Spain; h Centre Technique de Recherche et de Valorisation des Millieux Aquatiques (CITEB), c/o CYROI 2, Rue Maxime Rivière, 97490 Sainte Clotilde, La Réunion, France

**Keywords:** Ciguatoxins, Intercomparison exercise, Liquid chromatography, Mass spectrometry, Matrix effects, Toxin profile analysis

## Abstract

Ciguatoxins (CTXs) are marine biotoxins that can contaminate seafood and if consumed can result in ciguatera poisoning (CP). The analysis of CTXs is challenging, as they occur in complex tissue matrices, cause CP symptoms at trace amounts (<1 μg kg^−1^), and certified reference materials are not available. Currently, no standard operating protocols exist for sample preparation or instrumental analysis. Five laboratories worldwide participated in this first-time method comparison study, which used sample extracts containing different CTX groups (CTX4A group, CTX3C group, C-CTX-1) to identify factors impacting CTX quantitation using LC-MS/MS and LC-HRMS. Matrix effects were found to significantly influence CTX quantitation, along with factors such as instrument, eluents, or selected precursor ion. CTXs were quantified using commercially available, non-certified CTX1B and CTX3C standards. Analogues of the CTX groups behaved differently with regard to matrix effects and suitable calibrants with differences between laboratories exceeding a factor of 10 in some cases.

## Highlights

• Five laboratories worldwide participated in a collaborative study for CTX analysis.
• CTX analogues in six sample extracts were determined by LC-MS/MS and LC-HRMS.
• Varying matrix effects were observed, being instrument and CTX analogue dependent.
• The external calibrant used was a determining factor for CTX quantification.
• The study provides unprecedented insight into method variability for CTX analysis.

## 1 Introduction

Ciguatoxins (CTXs) are a group of marine biotoxins produced by benthic dinoflagellates of the genus *Gambierdiscus*, and possibly *Fukuyoa* (Murray et al., 2024; Satake et al., 1993; Satake et al., 1996) which can be biotransformed within the marine food web, mainly by oxidation (Ikehara et al., 2017; Li et al., 2020; Yogi et al., 2014) CTXs can cause ciguatera poisoning (CP) after oral consumption, 10,000 to 50,000 cases per year are estimated, however, CP is considered underreported (Friedman et al., 2017). The characteristic symptoms of CP are neurological disorders such as cold allodynia (reversal of the sensation of heat and cold) or cold dysesthesia (pain sensation at contact with cold materials or food), but a variety of gastrointestinal, cardiovascular, or neurological symptoms have been reported in the context of CP (Friedman et al., 2017).

**Fig. 1:**
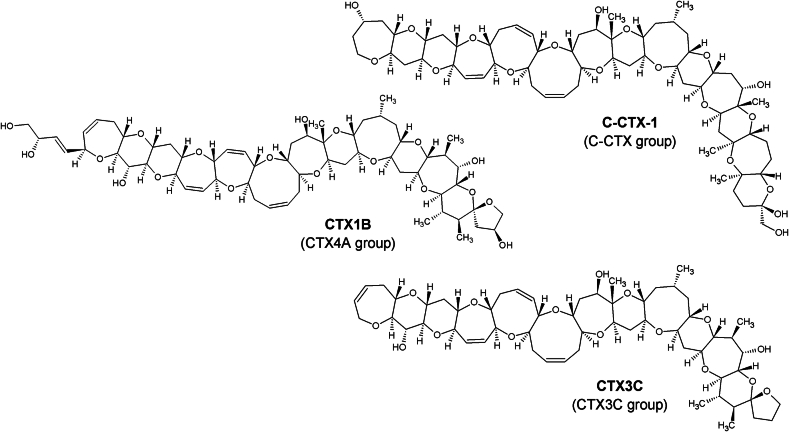
Structures of ciguatoxins representing the three different groups investigated in this study, stereochemistry according to FAO and WHO (2020).

CTXs are lipophilic and have been shown to accumulate within the marine food web, although the intricacies of the trophic transfer are still under investigation (Holmes & Lewis, 2022; Ledreux et al., 2014; Yogi et al., 2014). CTXs are divided into different groups based on their structure (Fig. 1), with more than 30 CTX analogues having been described (Estevez et al., 2023; FAO and WHO (2020); Mudge et al., 2023). Due to their complex polyether structure, the synthesis of CTXs requires multiple steps (e.g. CTX1B: 22 steps with an average yield of 73 % per step (Hamajima & Isobe, 2009)) and therefore, the approach to generate standards for analysis has been reliant upon extracted natural sources such as fish or microalgae. For the structural elucidation of CTX1B, 125 kg moray eel (*Gymnothorax javanicus*) viscera were used to isolate and purify 0.35 mg toxin (Murata et al., 1990). So far, only a few CTX analogues are commercially available, but not as certified standards or reference materials, although it is acknowledged that some of the isolated CTXs were quantified by quantitative nuclear magnetic resonance (qNMR) (Kato & Yasumoto, 2017).

Within the last two decades, several methods for the analysis of CTXs by LC-MS/MS and LC coupled to high resolution MS (HRMS) have been published, following different approaches for the sample preparation and the analysis (overviews provided by Harwood et al. (2017) and Pottier et al. (2023)). Methods differ regarding the tissue type (wet or dry), the sample weight or the solvent volume used for extraction (i.e., tissue equivalent (TE) per mL in the final extract or solvent volume per TE). There is a high variety in the number and design of the individual clean-up steps, with different solvents such as acetone, (aqueous) methanol or ethyl acetate often cited as being used for extraction. Most methods generally include steps for defatting the raw extract using *n*-hexane, usually followed by solid phase extraction (SPE) with normal and/or reversed phase sorbent material (Harwood et al., 2017; Pottier et al., 2023). The variety of preparation methods described leads to extracts with different matrix compositions and potentially different matrix effects, which can result in measurement uncertainties even when analyzed on the same analytical system. Such aspects can hamper the comparability of quantitative results.

For LC-HRMS and LC-MS/MS analysis, source geometry and parameters of the electrospray ionization (ESI) source often depend on the manufacturer of the analytical instrument and settings cannot be entirely compared and transferred between instruments of different companies. Parameters such as the pH of the eluents, the solvents utilized, eluent additives, the type of adduct and fragment selected for detection of the respective CTX analogues are chosen individually by each laboratory. All these aspects, individually or combined, can result in varying matrix effects observed for the same sample analyzed on different instruments or with different methods, which can lead to (significantly) different quantitative results. The lack of certified standards or reference materials adds further uncertainty in this context.

Collaborative studies rely upon large amounts of source material that can be equally divided among participants. Because CTX contaminated material is difficult to acquire in sufficient amounts, collaborative studies in the field of CTX analysis are rare, leading to a fundamental knowledge gap in CTX research regarding instrumental analysis and its variability and regarding comparability of quantitative results obtained by different laboratories. Therefore, a focused intercomparison study between five laboratories was initiated in the context of the RASCS project (Risk Assessment Strategies for Contaminants in Seafood (Diogène et al., 2023)) with the aim to acquire a first insight into the variability of LC-MS/MS and LC-HRMS methods applied to the same samples among different laboratories and their impact on the quantitative results. For this, sample extracts were shared among partners and for one sample matrix, two extraction methods and their impact on the quantitation and profile analysis were investigated. For quantitation, commercially available standards were shared among participants to exclude any impact of different standard lots on the quantitative results. The samples investigated were provided by several researchers worldwide. The samples included CTX analogues that were geographically representative for the main structural backbone diversity of CTXs and included the three major CTX groups (CTX4A group, CTX3C group, C-CTX-1).

Currently, for CTX, no standard operating protocols exist for sample preparation or instrumental analysis. Thus, no analytical parameters were defined, allowing participants to apply their individual methods including chromatographic conditions, ESI source parameters, or precursor and fragment ions used for detection which corresponds to the current approach (individual methods used by the respective laboratories). This is the first worldwide collaborative study in the field of CTX analyses at this scale to obtain a comprehensive understanding of the current status and potential hurdles facing the field of CTX analysis by mass spectrometric methods.

It is important to note that it was not the intention to recommend or reject any of the methods used by the participants in this study. The aim was to highlight unresolved questions in the analytical field of CTX research and provide elements of discussion as the research field works towards harmonizing a CTX analysis protocol. Certified reference materials and analytical standards were not available for this trial, thus, none of the contents provided within this study can be considered more or less accurate. Therefore, the strength of this study is the relative reporting among approaches applied among the laboratories and how the individual approaches influenced the reported values.

## 2 Material and methods

### 2.1 Standards

CTX1B (two times 100 ng, lot ESJ5851) and CTX3C (two times 100 ng, lots APK4222 and ESN0667) were obtained from FUJIFILM Wako Chemicals Europe GmbH (Neuss, Germany). Standards were received as dry residue in a glass vial. According to the manufacturer's safety data sheet, analogues were extracted from naturally incurred sources. No details concerning the compound purity were provided.

Each standard was reconstituted in 1 mL of methanol and solutions of the same analogue were combined to yield a final volume of 2 mL. For homogenization, the solution was vortexed for 30 s. For each analogue, an aliquot (200 μL) was transferred into a glass vial and stored at −20 °C. Before shipping, the volume was reduced to dryness under ambient conditions in the fume hood, small solvent residues were removed in a gentle stream of N_2_ at room temperature. The shipment of dried residues was considered appropriate as it corresponds to the procedure used by the manufacturer. After arrival at the collaborating laboratory, each standard was redissolved in 1 mL methanol (final analogue concentration: 20 μg L^−1^) and stored at −20 °C before usage.

Mixed standards for calibration curves were prepared in methanol following a uniform pipetting scheme among participants. In total, seven calibration levels were analyzed, ranging from 0.5 to 10 μg L^−1^ (Table S1). These concentrations were calculated based on the information provided by the manufacturer for the product (quantity of the analogue without purity). Throughout this study, the term “commercial standard concentrations” is used for these standard solutions. No matrix-matched calibration was performed, also due to the diversity of sample matrices analyzed (see Section 2.2).

Standards of CTX1B and CTX3C, quantified by qNMR, were kindly provided by Prof. Yasumoto (Japan Food Research Laboratories JFRL, Tokyo, Japan). An aliquot of each stock standard was diluted to 10 μg L^−1^ and used as working standard. The diluted qNMR standard and the 10 μg L^−1^ standard of the commercial standard (Table S1) were analyzed as triplicate, interspersed injections by the participant in New Zealand. Peak areas of both standards were compared to estimate the concentration of the commercial CTX analogues (data provided in Table S2). The term “standards with qNMR adjusted concentrations” is used within this study for standards whose concentrations were re-calculated based on the obtained results.

### 2.2 Sample material

Fish samples of different geographic origins containing several CTX profiles were investigated (Table 1). All samples were naturally incurred and no external standards were added to the material prior extraction or the final extracts. In addition to instrumental analysis, and to guide the selection of samples, CTX-like toxicity had been evaluated by the *in vitro* cell-based assay using mouse neuroblastoma cells (N2a-bioassay) adapted from Manger et al. (1993). All quantitative results are provided in μg kg^−1^.

**Table 1:** Type and origin of samples used within the intercomparison study.

Sample	Species	Origin	TE in final extract
1 (A-C)	*Lutjanus bohar* (Two spot red snapper)	kindly provided by Centre Technique de Recherche et de Valorisation des Millieux Aquatiques (CITEB) (La Réunion, France) origin: La Réunion, France (Mont Laperousse bank) species assignment based on morphological observation	A^1^: 10 g mL^−1^ B^1^: 4 g mL^−1^ C^2^ (fractionated): 5 g mL^−1^
2 (A-C)	*Seriola rivoliana* (Longfin yellowtail)	kindly provided by Institute of Animal Health and Food Safety (IUSA), University of Las Palmas de Gran Canaria (ULPGC) (Spain) origin: Tenerife (Canary Islands, Spain) linked to CP outbreak on Canary Islands in 2019 (see Table 3 in Varela Martínez et al. (2021)) species assignment based on morphological observation	A^1^: 10 g mL^−1^ B^1^: 4 g mL^−1^ C^2^ (fractionated): 5 g mL^−1^
3, 4, 5	*Plectropomus laevis* (Blacksaddle Coral grouper)	kindly provided by Cawthron Institute (Nelson, New Zealand) origin: import from Fiji linked to CP outbreak in New Zealand in 2020 (Murray et al., 2021) samples provided as meal remnant (fish curry homogenate, 3) or fried fish fillet (4 and 5); all samples originated from the same fish species confirmed by DNA barcoding	3^1^: 10 g mL^−1^ 4^1^: 4 g mL^−1^ 5^3^ (undiluted): 30 g mL ^−1^
6	*L. bohar*	kindly provided by German Federal Institute of Risk Assessment (Berlin, Germany) origin: import from Vietnam and India linked to CP outbreak in Germany and Netherlands (Loeffler et al., 2022; Loeffler et al., 2023) species confirmed by DNA barcoding	6^1^: 5 g mL^−1^

TE – tissue equivalent, sample preparation according to ^1^Spielmeyer et al. (2021), ^2^Spielmeyer et al. (2024), ^3^Murray et al. (2018).

For samples prepared according to Spielmeyer et al. (2021), the method generates two fractions in the final sample preparation step (filtrate and eluate). Only the eluates were used within this study as this fraction contains polar CTX analogues and a portion of CTX3C. Depending on the sample matrix, different sample weights were used for extraction (Table 1).

For samples 1 and 2, an aliquot of the samples 1 A and 2 A was fractionated by SPE, using a normal phase (silica) SPE (Spielmeyer et al., 2024), generating samples 1 C and 2 C. For each sample, the fractions showing the respective CTX analogue in the LC-MS/MS chromatogram and toxicity in the N2a-bioassay (performed according to Loeffler et al. (2021)) were combined.

Sample 5 (same matrix as sample 4) was prepared according to Murray et al. (2018) as a bulk extract of fried fish fillet (naturally contaminated material, 120 g). An aliquot of 1 mL was sent from New Zealand to Spain and distributed from there to the other participants.

For all samples, a defined aliquot was transferred into a glass vial and reduced to dryness as described in Section 2.1. Aliquots ranged from 70 to 100 μL, depending on the amount of sample material available for extraction. After arrival at the participants, the samples were redissolved in methanol using the defined volume (70 to 100 μL) and stored at −20 °C before usage. For analysis, sample 5 was diluted 1:10 with methanol by each participant, corresponding to 3 g TE mL^−1^ used for injection.

### 2.3 Matrix effects

Matrix effects were determined for freeze-dried *Caranx* spp. fillet (*C. ruber, C. latus*, sample origin: Saint François, Guadeloupe). *Caranx* species can be involved in CP and C-CTXs can be isolated from *C. latus* (Pottier et al., 2002), making this matrix a realistic scenario in the context of CTX analysis. The samples were prepared according to Spielmeyer et al. (2021) and only the eluates were used for analyses. The final extract corresponded to 1 g dry TE mL^−1^. Extracts showed no CTX-like toxicity in the N2a-bioassay (conducted according to Loeffler et al. (2021) with a detection capability at 0.0048 μg CTX3C eq. per kg) and thus, were considered as blank material. An aliquot of 400 μL was transferred into a glass vial and reduced to dryness as described in Section 2.1. After arrival at the collaborating laboratory, the sample was redissolved in 400 μL methanol and stored at −20 °C before usage.

For analysis, 20 μL CTX1B and 20 μL CTX3C standard solution (20 μg L^−1^ each) were mixed with either 160 μL blank extract or methanol, resulting in a final concentration of 2 μg L^−1^ for each analogue. Both matrix sample and matrix free standard were analyzed in triplicate. The matrix effect was expressed in the form of a bias (signal suppression enhancement, SSE), based on Eq. 1. A positive value corresponds to a signal enhancement, a negative value to a signal suppression.

(1) $$\text{matrix effect}\;\left[\%\right]=\left(\frac{{\text{peak area}}_{\text{matrix sample}}}{{\text{peak area}}_{\text{matrix free standard}}}*100\%\right)-100\%$$

### 2.4 LC-MS/MS and LC-HRMS analysis

Sample analyses were conducted using the participants' internally established methods. LC and MS method parameters of the respective laboratories are provided in Table 2, highlighting some differences between the laboratories in bold. It is worth mentioning that laboratory A was the only partner using an HR mass spectrometer. Laboratory E used basic eluents whereas the other participants used acidic conditions (Table 2).

**Table 2:** LC- and MS-method parameters utilized by the respective laboratories.

Institute parameter	Laboratory A			Laboratory B			Laboratory C			Laboratory D			Laboratory E		
*LC method*															
LC system	Thermo MS Surveyor			Thermo Scientific Vanquish Horizon			Agilent 1290 Infinity II			Waters Acquity UPLC I-class			Waters Acquity UPLC I-class		
Eluents	A: 2 mM ammonium formate in water +0.1 % formic acid			A: 5 mM ammonium formate and 0.1 % formic acid in water			A: 1 mM ammonium acetate in water +0.5 % formic acid			A: 2 mM ammonium formate in water +0.1 % formic acid			A: water +**0.2 % (*v/v*) ammonium hydroxide**		
	B: acetonitrile/water 95:5 (*v/v*) with 2 mM ammonium formiate +0.1 % formic acid			B: **methanol**			B: **methanol/ acetonitrile 3:1 (*v/v*)**			B: acetonitrile/water 95:5 (*v/v*) with 2 mM ammonium formiate +0.1 % formic acid			B: acetonitrile/water 95:5 (*v/v*) + **0.2 % (*v/v*) ammonium hydroxide**		
Gradient	time [min]	%B	flow [mL min^-1^]	time [min]	%B	flow[mL min^-1^]	time [min]	%B	flow [mL min^-1^]	time [min]	%B	flow [mL min^-1^]	time [min]	%B	flow [mL min^-1^]
	0	50	0.25	0	82	0.40	0	78	0.45	0	5	0.40	0	5	0.55
	1.0	50	0.25	1.0	82	0.40	10.0	92	0.45	1.0	50	0.40	1.0	5	0.55
	9.0	90	0.25	15.0	100	0.40	10.1	99	0.45	5.0	100	0.40	3.5	50	0.55
	9.1	100	0.25	15.5	100	0.80	10.6	99	0.60	7.0	100	0.40	7.5	75	0.55
	12.0	100	0.25	19.0	100	0.80	13.0	99	0.60	7.1	5	0.40	8.0	95	0.55
	14.0	50	0.25	19.5	82	0.40	13.2	78	0.45	11.0	5	0.40	9.0	95	0.55
	25.0	50	0.25	23.0	82	0.40	16.0	78	0.45				9.2	5	0.55
													10.0	5	0.55
Column	Thermo Hypersil Gold C18 (100 × 2.1 mm, 1.9 μm)			Agilent Poroshell 120 EC-C18 (100 × 2.1, **2.7 μm**)			Phenomenex Gemini NX-C18 (**150** × 2 mm, **3** μ**m**)			Waters Acquity UPLC BEH C18 (**50** × 2.1 mm, 1.7 μm)			Waters Acquity UPLC BEH **phenyl** column (100 × 2.1 mm, 1.7 μm)		
Column temperature	**25 °C**			40 °C			40 °C			40 °C			**50 °C**		
Injection volume	**5** μ**L**			2 μL			2 μL			2 μL			2 μL		
*MS method*															
MS system	Thermo Exactive HCD-Orbitrap			Thermo Scientific TSQ Altis (Triple-Quadrupole)			Sciex Qtrap 6500+ (Triple-Quadrupole)			Waters Xevo TQ-XS (Triple-Quadrupole)			Waters Xevo TQ-S (Triple-Quadrupole)		
resolution	**high (set to 50.000, FWHM, *m/z* 200)**			low			low			low			low		
Mode of analysis	**Full Scan** (400–1500 *m/z*)			MRM / SRM			MRM / SRM			MRM / SRM			MRM / SRM		
Ionization	H-ESI positive			ESI positive			ESI positive			ESI positive			ESI positive		
Ion Spray Voltage	4000 V			4500 V			5500 V			3000 V			3500 V		
Interface Temperature	300 °C (Heater) 275 °C (Capillary)			375 °C (Vaporizer) 300 °C (Capillary)			500 °C (Ion Source)			450 °C (Desolvation) 150 °C (Ion Source)			600 °C (Desolvation) 150 °C (Ion Source)		
Gases	35 psi (Sheath) 10 psi (Aux) 0 psi (Sweep)			35 arb (Sheath) 15 arb (Aux) 3 arb (Sweep)			70 psi (Gas 1 / 2) 40 psi (Curtain)			1000 L/h (Desolvation gas flow) 150 L/h (Cone gas flow) 7.0 bar (Nebuliser)			1000 L/h (Desolvation gas flow) 150 L/h (Cone gas flow) 7.0 bar (Nebuliser)		
Collision cell gas				Argon (1.5 mTorr)			Nitrogen (medium)			Argon (0.15 mL min^−1^)			Argon (0.15 mL min^−1^)		
Needle position (ESI Source)	0 / 1 / C (x / y / z)			0 / M / 1.2 (horizontal / depth adjustment / front-to-back)			5.0 / 5.0 (vertical / horizontal)			7.0 / 6.5 (vertical / horizontal)			−0.84 / 2 (*Z*-offset / vertical)		
Additional parameters	AGC target: Balanced (1e6) Maximum inject time: 250 msec Capillary voltage: 47.5 V Tube lens voltage: 186 V Skimmer voltage: 18 V														
Software for data aquisition	Thermo Xcalibur 3.0.63			TraceFinder™ 4.1 - EFS			Analyst 1.6.3			MassLynx V4.2			MassLynx V4.1		
Software for data evaluation	Trace Finder 5.1			TraceFinder™ 4.1 - EFS			SciexOS-Q 3.0			TargetLynx XS software			TargetLynx V4.1		

FWHM - full width at half maximum; H-ESI – heated electrospray ionization; some major methodological differences between the participants are highlighted in **bold.**

Calibration standards were analyzed with ascending concentration, followed by a methanol injection, the investigated samples, and another methanol injection. This sequence was repeated two more times to deliver triplicate injections for each calibration standard and sample (Table S3). The CTX contents of the samples were calculated using the mean calibration function established from the three repetitions. A linear regression without any weighting was applied. Details concerning the monitored CTX analogues as well as the extracted ion traces or respective ion transitions are provided in the Supporting Information (Table S4 and S5). Information on the quantifier ions is included in Table S5.

### 2.5 Statistical analysis

For intra-laboratory comparison, statistical analyses were performed using the *t*-test function in Microsoft Office Excel 2021. Results with *p* < 0.05 were considered significantly different. For inter-laboratory comparison, a One Way ANOVA was applied using SigmaPlot 14.0. In all cases, data of triplicate injections (n = 3) were applied to the tests. Data sets were checked for and fulfilled conditions of normality (Shapiro-Wilk test) and equal variances (Brown-Forsythe test). In the case of significant differences between the groups, a pairwise comparison of all laboratories was conducted using the Tukey post-hoc test. Results with *p <* 0.01 were considered significantly different. Graphics were created using SigmaPlot 14.0.

## 3 Results and discussion

### 3.1 Calibration curves

**Fig. 2:**
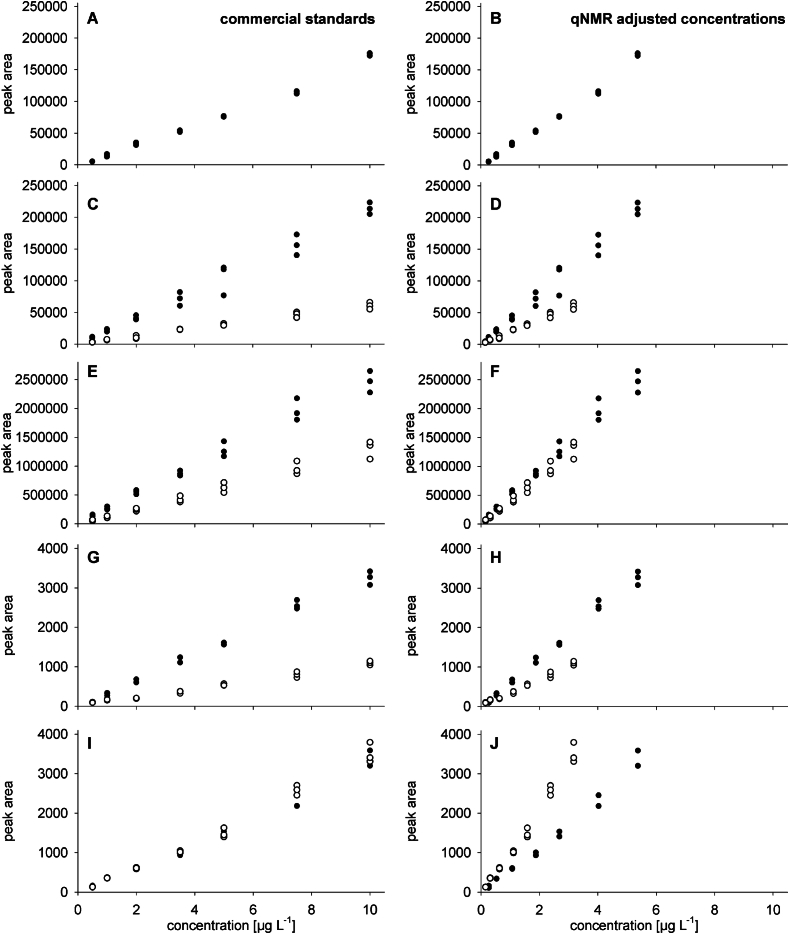
Calibration curves obtained for CTX1B (solid symbol) and CTX3C (open symbol) by **(A,B)** Lab A, **(C,D)** Lab B, **(E,F)** Lab C, **(G,H)** Lab D, **(I, J)** Lab E; panels show the curves for the commercial standards' concentration (left) or standards with qNMR adjusted concentration (right); data points show results of triplicate injections (except Lab D and 5 μg L^−1^ standard for CTX3C: *n* = 2); scaling of the axes was kept constant between the left and right panels to better illustrate the impact of the concentrations on the slope of the calibration functions.

Correlation coefficients (R^2^) of the calibration curves were > 0.975 with only one exception (R^2^ = 0.941) (Table S6, Fig. 2). Measurement precision ranged between 1.0 and 33.2 % without specific trends for the individual analogue, concentration, or setup (Table S7). For the HRMS instrument (Lab A), no data for the CTX3C calibration could be provided as the concentrations investigated were below the LOD of the instrument (11 μg L^−1^).

For Labs B to E, differences were observed for the slopes of the CTX1B and CTX3C calibration functions, depending on the analytical setup and the reference used for the calculation of the standard concentration (Fig. 2, Table S6). For the commercial standards, Labs B, C, and D showed a lower sensitivity for CTX3C than for CTX1B (factor 2–3.3) which might be due to different responses of the analogues in general or on the respective systems only. In contrast, almost identical slopes were obtained for both analogues for Lab E. For the standards with qNMR adjusted concentrations, Labs B and D still showed a lower sensitivity for CTX3C than for CTX1B (factor 2), whereas Labs C and E showed a 10 % lower and 67 % higher sensitivity for this CTX analogue compared to CTX1B, respectively (Fig. 2, Table S6).

All laboratories used solvent additives. In the case of Labs A, D, and E, the additive concentration was kept constant during the gradient elution (i.e., same additives in the aqueous and organic solvents). Labs B and C used no additives in the organic solvent (Table 2). Here, the acidity of the eluent changes within the analytical run which might influence ionization efficiencies (i.e., potentially better ionization with higher acidity at the time of elution). This change might be another reason for the lower sensitivity observed for CTX3C in comparison to CTX1B for these two laboratories. However, also for Lab D different slopes were observed for the two analogues, although eluent additives are kept constant within the analytical run (Fig. 2, Table 2, Table S6). The most profound difference between Labs B, C, D and Lab E was the use of basic eluents by Lab E, the only partner using basic mobile phases in this study. It appears that the basic conditions have a different effect on the ionization of each CTX analogue, when comparing matrix free standard solutions, leading to a similar or even higher slope for the CTX3C calibration (Fig. 2, Table S6). It should be investigated in future studies, if this effect of the eluents' pH is specific to this instrument only or if a similar observation is made when using instruments from other manufacturers.

Different sensitivities for CTX1B and CTX3C can have a major impact on the quantitation of CTX analogues for which no commercial standards are available. Reported contents can differ considerably based on the standard used for quantitation, both within one laboratory and between laboratories. The commercially available CTX analogues are sold as non-certified standards, and this adds further uncertainty to the quantitation. The CTX1B and CTX3C standards shared within this study were extracted from naturally incurred sources, according to the safety data sheet provided by the manufacture. The sample weight on the label was provided without details concerning the compound purity. These standards were checked against qNMR standards which were most likely extracted from naturally incurred sources as well (according to information provided in Kato and Yasumoto (2017)). The commercial standards revealed a reduced signal intensity, showing 54 % and 32 % of the qNMR standards' peak areas, respectively (Table S2). Aliquots of the commercial standards were reduced to dryness before shipment (see Section 2.1) to avoid potential losses due to leaking during transport. Part of the analyte might not have been redissolved in methanol, e.g., due to sorption of the analyte on the glass surface. So far, sorption of CTXs was only reported on plastic surfaces (Lewis et al., 2009). Due to the chemical stability of CTXs (e.g., heat stability (Abraham et al., 2012; Pottier et al., 2002)), a degradation of the analogues during non-cooled transport appears less likely. Neither standard (commercial or qNMR) is certified and therefore, no final conclusion can be drawn as to which concentration offers the most accurate quantitative results. The impact of different standard concentrations and different sensitivities of the respective references on the CTX quantitation is discussed in detail in Section 3.3.

### 3.2 Matrix effects

All participants obtained an aliquot of a blank matrix extract from *Caranx* spp. which was spiked with CTX1B and CTX3C at 2 μg L^−1^ on the day of analysis. Peak areas of the spiked matrix extract were compared to a matrix free standard solution prepared in the same way using methanol (Section 2.3). Matrix effects ranged from −32 % to +8 % for CTX1B and from −72 % to +115 % for CTX3C (Table S8). Labs A (+8 %) and D (+5 %) reported almost no matrix effects for CTX1B, with only slight signal enhancements, whereas Labs B, C, and E obtained a signal suppression of −32 % to −23 %. Matrix effects were greater for CTX3C, as Lab B showed a pronounced signal enhancement (+115 %), while a signal suppression was described by the other participants ranging from −10 % (Lab C) over −25 % (Lab D) down to −72 % (Lab E) (Table S8). This implies that interfering compounds, which cause ionization competition with CTXs, were still present in the extract. However, their impact on the signal intensity was variable, depending on the setup utilized for analysis (e.g., ionization source parameters, eluent composition, proportion of organic eluent at the time of elution). Based on these results, matrix effects reported for a specific method should be evaluated with caution, as low matrix effects, determined for a single setup, might not correspond to a high extract purity.

Recovery rates are determined by spiking a blank matrix before sample preparation for estimating the analyte recovery during extraction, clean-up, and analysis and, therefore, matrix effects are included in this parameter. For the same sample, Labs A to E would have determined different recovery rates due to different matrix effects on their respective instruments. Therefore, the extraction efficiency of sample preparation methods should be determined and subsequently provided. For this parameter, the recovery rate is corrected by the matrix effect (Caban et al., 2012). The extraction efficiency allows an estimation of the analyte recovery from a specific matrix, independent of the matrix effect occurring on the instrument utilized for analysis. Therefore, the extraction efficiency parameter enables a better comparability of sample preparation methods reported in the literature, especially if no reference method is available as in the case of CTX analysis. For the matrix samples investigated in this study, no correction by recovery rate or matrix effects was performed, also as different matrices were investigated and additional CTX analogues were quantified for which no matrix effects could be evaluated due to the lack of available standards.

### 3.3 Qualitative and quantitative analysis

In the following sections, the laboratories' results regarding the qualitative and quantitative analysis are discussed. This is the first detailed intercomparison study for the CTX analysis by LC-MS/MS and LC-HRMS. Thus, no detailed evaluation of the data obtained within this trial can be made in the wider context of previously published data.

For samples 1 and 2, several factors were considered such as the extract purity, external standard, or the ion transitions used for quantitation (Section 3.3.2). For samples 3, 4, and 5, two extraction methods were compared, assessing their impact on quantitation and profile analysis (Section 3.3.3). The aspects of qualitative analysis and peak identification were most prominent for the CTX3C group analogues present in sample 6, with commercial standards not being available for most compounds (Section 3.3.4).

#### 3.3.1 Qualitative analysis

The participants identify peaks based on the retention time, either by comparison with an external standard or reference material or elution profiles that were reported in peer reviewed publications (Yasumoto et al., 2000; Yogi et al., 2011). In addition, several approaches are used for peak identification. For the LC-HRMS analyses (Lab A), the signals of potential CTX analogues have to meet the following criteria: peak area > 1e+03; retention time ± 0.5 min; mass accuracy window <10 ppm; match of experimental versus theoretical isotopic pattern >50 %. The signals identified in the samples of this study accomplished these criteria with a mass accuracy of 1 to 8.6 ppm, and 52 to 100 % for isotopic profile match. Other possible CTXs were detected that did not meet some of these criteria (mass accuracy, isotopic profile) and without standards for these potential CTX analogues, these signals were not included by Lab A.

For low-resolution analyses and the monitoring of the pseudo ion transition of the sodium adduct (Labs B and C), potential CTX peaks are further investigated by analyzing other precursor ions ([M+H-H_2_O]^+^, [M+H]^+^, [M+NH_4_]^+^) and their fragments due to their higher selectivity (e.g., loss of water molecule(s), 108.9, 191.1 for C-CTX-1 (Kryuchkov et al., 2020)). Different methods are used for these additional analyses (e.g., lower source temperature for improving adduct intensity) which are partly conducted on different instruments (including high resolution). In routine analysis, the pseudo-ion transition is used due to its higher signal intensity and because it does not require any information about the fragmentation pattern to configure the method. This is particularly useful for analogues that are not available as standards or reference material.

Labs D and E (that use instruments of the same manufacturer and acidic and basic mobile phases, respectively) investigate precursor ions that can easily fragment ([M+H-H_2_O]^+^, [M+H]^+^, [M+NH_4_]^+^), resulting in two or three fragments which are recorded for each compound (Table S5). The ratio of the MRM transitions is checked and variations of less than 30 % (Lab D) or 5 % (Lab E) are accepted, compared to available reference materials and standards.

#### 3.3.2 Quantitation of the CTX analogues CTX1B and C-CTX-1 – Impact of extract purity (samples 1, and 2)

The fish specimens for samples 1 and 2 were prepared in three different ways (Table 1, Section 2.2) to investigate the impact of the extract purity on the quantitation within one lab, and the comparability of the results obtained by the different laboratories. Each matrix contained one known CTX analogue, either CTX1B or C-CTX-1, which were identified by all participants. Quantitative data (mean and standard deviation) of all participants are provided in detail in Table S9.

**Fig. 3:**
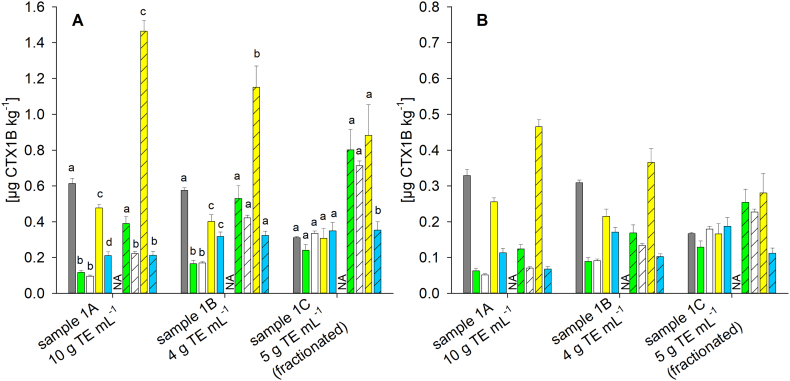
Quantitative results obtained for sample 1 depending on the sample preparation (see Table 1 and Section 2.2 for details) and the laboratory (from left to right Lab A (grey), B (green), C (white), D (yellow), E (blue)) with quantitation based on the calibration functions obtained for **(A)** the concentration of the commercial standard and **(B)** the qNMR adjusted concentrations; bars represent the quantitative results obtained via the calibration of CTX1B (blank) and CTX3C (shaded); results are provided as mean, error bar shows standard deviation (*n* = 3); NA – no data available for Lab A and the CTX3C calibrant as the concentration of the standards was <LOD (11 μg L^−1^); in panel A, different letters for each sample and calibrant represent a significant difference between the laboratories after pairwise comparison (One Way ANOVA, Tukey post hoc test, *p <* 0.01), letters are not provided in panel B to avoid redundancy; the reader is referred to the y-axis in panel B showing half the dimension compared to panel A; for detailed data see also Table S9. (For interpretation of the references to colour in this figure legend, the reader is referred to the web version of this article.)

CTX1B content estimates in sample 1 were dependent on the extract preparation and reference standard used for quantitation (Fig. 3). For sample 1 A, when using the CTX1B calibrant, contents aligned with the results from the matrix effects study. Labs A and D quantified significantly higher levels than the other participants (*p <* 0.01), with the highest and lowest average values differing by a factor of six. For the matrix effects and CTX1B, Labs A and D showed a small signal enhancement, whereas Labs B, C, and E reported signal suppression (Table S8).

Reducing the matrix load in the extract, by using less material for extraction (lower TE mL^−1^), led to a convergence of the results as reflected by quantified levels in sample 1 B (difference of a factor by 3.5 between the highest and lowest average values). The comparability was further improved by fractionation using normal phase (silica) SPE and, thus, an orthogonal separation mechanism (sample 1 C, difference of a factor by 1.5) (Fig. 3). For sample 1 C, and the CTX1B calibrant, no significant differences were observed among the participants. This underlines the impact of sample purity on the comparability of quantitative results.

Sample 1 C could be considered as an ideal case, with similar contents determined by several laboratories using their individual analytical methods and using the respective analogue as a calibration standard (i.e., CTX1B standard calibration for CTX1B quantitation). However, uncertainties concerning the ‘true content’ remain. Contents determined using the standards with qNMR adjusted concentrations were approximately a factor of 2 lower than contents calculated based on the commercial standard concentrations (Fig. 3, Table S9).

When the CTX3C calibrant was used for CTX1B quantitation in sample 1 C, results from Labs B, C and D were similar (Fig. 3). However, the contents were significantly higher than those determined via the CTX1B calibration (*p <* 0.05 for intra-lab comparison), reflecting variable instrument sensitivities for CTX1B and CTX3C on the respective instruments and methods applied (Fig. 2, Table S6). The extent of the discrepancy between the contents determined by calibration via either CTX1B or CTX3C depended on the assumed concentration of the calibration standards. For the commercial standard concentrations, contents for the CTX3C calibrant were 2 to 3 times higher whereas contents differed by a factor of 1.3 to 2.0 for the standards with qNMR adjusted concentrations (Fig. 3, Table S9). For Lab E and the uncorrected concentrations, the calibration functions of two analogues showed similar slopes and consequently, almost identical CTX1B contents were obtained, irrespective of the calibrant used (*p >* 0.05) (Fig. 3). However, for the qNMR adjusted concentrations, the CTX3C calibrant delivered a significantly lower CTX1B content in sample 1 C (factor 0.6, *p <* 0.05; Fig. 3). Among all laboratories, the lowest and highest contents in sample 1 C differed by a factor of 2.5 for the CTX3C calibrant (in contrast to factor 1.5 for the CTX1B calibrant).

The US Food and Drug Administration (FDA) provided a guidance value for CTX1B (0.01 μg CTX1B eq. kg^−1^). In all cases, this guidance value would have been exceeded, independent of laboratory, sample purity, calibration standard, or standard concentration's adjustment (Fig. 3). Within the European Union, products containing CTXs are not to be placed on the market (Commission Implementing Regulation (EU) 2019/627). Thus, in term of non-compliance, every positive confirmation of CTX would lead to an exclusion from the market, making the quantification of CTX a gratuitous discussion. However, if results of different laboratories should be compared, e.g., in the context of monitoring studies, or guidance values should be derived from epidemiological data, consensus must be found on how CTX quantification should be performed.

For sample 1 and CTX1B, the discussion about different calibrants (i.e., which CTX analogue should be used as reference) is more theoretical as CTX1B is a commercially available standard. The fact of missing analytical standards for quantitation becomes evident for C-CTX-1 in sample 2. For this analog, quantitation had to be performed using CTX1B and CTX3C, with both analogues belonging to structurally different CTX groups (CTX4A and CTX3C group, respectively), as no commercial standard is available for any C-CTX group analogue.

**Fig. 4:**
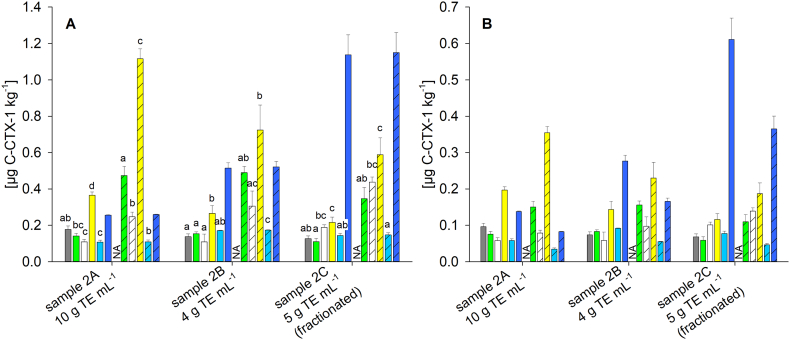
Quantitative results obtained for sample 2 depending on the sample preparation, the laboratory (for colour code see Fig. 3), and in the case of Lab E (blue bars) the ion transition used for quantitation with the loss of water ([M+H-H_2_O]^+^ / [M+H-3H_2_O]^+^, light blue) and the pseudo ion transition ([M+Na]^+^/[M+Na]^+^, dark blue); quantitation based on the calibration functions obtained for **(A)** the concentration of the commercial standard and **(B)** the qNMR adjusted concentrations; bars represent the quantitative results obtained via the calibration of CTX1B (blank) and CTX3C (shaded); results are provided as mean, error bar shows standard deviation (*n* = 3); NA – no data available for Lab A and the CTX3C calibrant as the concentration of the standards was <LOD (11 μg L^−1^); different letters for each sample and calibrant represent a significant difference between the laboratories after pairwise comparison (One Way ANOVA, Tukey post hoc test, *p <* 0.01), letters are not provided in panel B to avoid redundancy; results for Lab E and the sodium adduct were not included in statistical data evaluation; the reader is referred to the y-axis in panel B showing half the dimension compared to panel A; for detailed data see also Table S9. (For interpretation of the references to colour in this figure legend, the reader is referred to the web version of this article.)

The pattern of the contents in sample 2 A did not follow the same trend as in sample 1 A (Fig. 3, Fig. 4), indicating different matrix effects for CTX1B and C-CTX-1 on the respective systems used for analysis. Matrix effects of C-CTX-1 could not be investigated in this study due to the absence of standard material. Lab D determined the highest C-CTX-1 content for all samples. With increasing extract purity, quantitative results of Labs A to E (light blue bar for Lab E, Fig. 4) became more similar as observed for sample 1; however, significant differences were observed also for the cleanest extract (sample 2 C, *p <* 0.01) (Fig. 4). The impact of the different standard concentrations (uncorrected and qNMR adjusted values) followed the same trend as observed for sample 1. For most samples, contents determined via the CTX1B and CTX3C calibrant were significantly different (intra-laboratory comparison, *p* < 0.05; only sample 2 C for Labs C and D, *p* > 0.05). The US FDA provided guidance value for C-CTX-1 (0.1 μg C-CTX-1 eq. kg^−1^) would not have been exceeded in all cases, depending on the standard concentration's adjustment (Fig. 4, Table S9). Concerning food safety and consumer protection, it should be clarified how quantitative results that fall below the established guidance values are to be handled. A guidance value does not correspond to a limit value and all samples were proven as ‘C-CTX-1 positive’. As mentioned for sample 1, within the EU, products containing CTXs are not to be placed on the market (Commission Implementing Regulation (EU) 2019/627) independent on the CTX content. Here, the concentrations' adjustment would not be relevant for the non-compliance assessment.

For CTX analysis, different adducts and ion transitions regarding qualitative and quantitative analyses are used by different laboratories, depending on the signal intensity obtained on the respective instruments. In this study, three peaks were used for the HRMS (orbitrap) setup, resulting from the molecular ion, the ammonium and the sodium adducts. For the MS/MS (triple quadrupole) methods, the pseudo ion transition for the sodium adduct, fragments of the ammonium adduct, the molecular ion or the molecular ion after water loss were monitored by the participants (Table S5). Matrix effects could vary for different precursor ions (Sibat et al., 2018) and such variations could be relevant both within one sample (e.g., no matrix effect on the ammonium, but on the sodium adduct) and between samples with different extract purities (e.g., changing signal intensity for the sodium adduct, but not for the ammonium adduct). Both aspects were observed for sample 2. For example, the pseudo ion transition of the sodium adduct was used by Labs B, C, and E. Although it is not as specific or selective as a molecular ion transition detecting distinct fragments, it is often selected for CTX quantitation due to its high signal-to-noise ratio (Estevez et al., 2019; Yogi et al., 2011). For Lab E, a remarkable increase of the C-CTX-1 content in sample 2 B (factor 2.0) and sample 2 C (factor 4.4) was observed compared to sample 2 A (dark blue bars, Fig. 4), while no such increase was observed for Labs B and C. The basic eluent used by Lab E likely has a different impact on the formation of the sodium adduct of C-CTX-1 than the acidic conditions used by Labs B and C, resulting in the sharp increase of C-CTX-1 content with increasing extract purity in the case of Lab E. In addition, different MS instruments are used by the respective participants which might cause further variations.

Lab E also recorded a selective water loss ion transition ([M+H-H_2_O]^+^ / [M+H-3H_2_O]^+^) and here almost constant C-CTX-1 contents were found for the three samples (light blue bars, Fig. 4). The C-CTX-1 contents showed a comparable pattern to CTX1B contents reported by Lab E for samples 1 A/B/C (Fig. 3) with only a moderate increase, when extract purity was improved. Also, in the context of the data provided by the other participants, the contents obtained by Lab E for the water loss ion transition are considered more reasonable than the results of the pseudo ion transition. These results provide evidence that different precursor ions can undergo different matrix effects both within one sample and between samples. Without reference materials being available or method comparison studies such aspects are difficult to evaluate and this emphasizes the need for such materials and studies in the CTX research field. Based on the results reported here, it should be considered to monitor several ion transitions, if strong signal suppressions or enhancements are observed for a distinct ion transition e.g., with changing extract purities, to reduced unintended bias.

No commercial standards are available for the entire C-CTX group, therefore, matrix effects or the impact of eluent conditions on ionization for C-CTX analogues are difficult to evaluate. Several groups analyzing C-CTX-1 published chromatograms showing a broad peak for this compound (Mudge et al., 2023; Spielmeyer et al., 2024; Tudó et al., 2022). The issue of peak broadening might be due to fast epimerization and could be overcome by derivatization (Kryuchkov et al., 2020). This highlights a distinct difference between C-CTX-1 (and potentially the entire C-CTX group) and analogues of the CTX3C and CTX4A groups, for which no comparable issues have been reported. Consequently, quantitation of C-CTX group analogues might be more susceptible to analytical conditions than analogues of other CTX groups and this aspect could hamper comparability of C-CTX-1 values reported in the literature.

#### 3.3.3 Extraction method effects on quantitation and profile analysis of CTX4A group analogues (samples 3, 4, and 5)

The congeners CTX1B, 54-deoxyCTX1B, and 52-*epi*-54-deoxyCTX1B were identified by all participants in samples 3, 4, and 5. In this section, only the results obtained via the CTX1B calibration of the commercial standard concentrations are discussed in detail. Standard concentrations calculated based on the data provided by the manufacturer were chosen as qNMR standards are not available in all laboratories and, thus, the commercial standard approach was considered as the most common scenario for other research groups. Differences between quantitative data of the CTX1B and CTX3C calibrations followed the same trends as observed for samples 1 and 2 (Section 3.3.2), emphasizing the impact of the calibrant on the quantitation and comparability of the data between laboratories. As all detected analogues belonged to the CTX4A group, results obtained via the CTX1B calibrant were considered as more appropriate than contents calculated for the CTX3C calibrant (see also discussion in Section 3.3.4). For the profile analysis, the calibrant selected had negligible impact on the ratio of the congeners. Data obtained using standards with qNMR adjusted concentrations as well as the comparison of the CTX1B and CTX3C calibrant are provided in the Supporting Information (Fig. S1, Fig. S2, Table S10).

The material used to generate samples 3, 4, and 5 were from the same fish, which originated from a CP event in New Zealand (Section 2.2, Table 1). Sample 3 was a fish curry meal remnant, whereas samples 4 and 5 were fried fish fillet. The extracts of samples 3 and 4 were prepared according to the protocol described in Spielmeyer et al. (2021), with a slight modification for sample 4 (sample weight for extraction was 2 g). Sample 5 was extracted according to Murray et al. (2018). Differences in the extraction and clean-up protocols are reflected by the quantitative results.

**Fig. 5:**
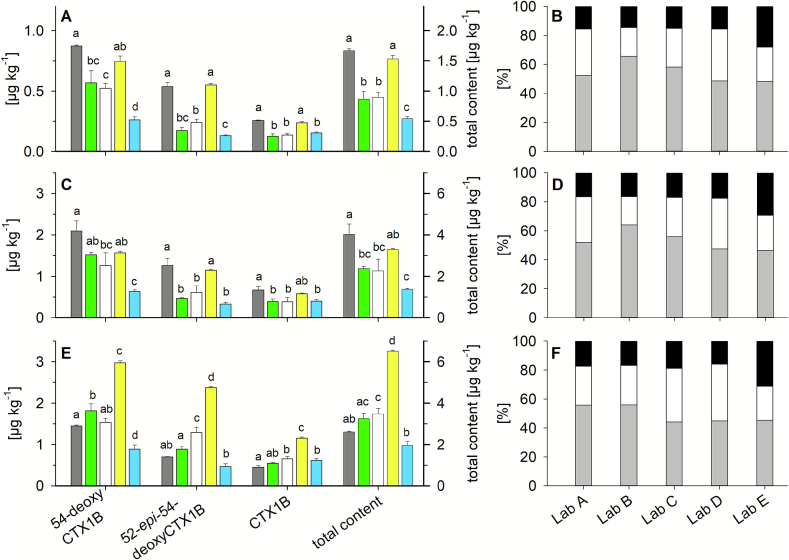
Quantitative (left, in colour) and profile analysis results (right, grey scale) obtained for **(A,B)** sample 3 (fish curry, meal remnant), **(C,D)** sample 4 (fried fish fillet), **(E,F)** sample 5 (fried fish fillet); samples 3 and 4 were prepared according to (Spielmeyer et al., 2021), sample 5 was prepared according to (Murray et al., 2018) (see Table 1 and Section 2.2 for further details); bars in the left panels represent (from left to right) the quantitative results obtained by Lab A (grey), B (green), C (white), D (yellow), and E (blue) for the CTX1B calibrant; results are provided as mean, error bar shows standard deviation (*n* = 3); bars in the right panels represent profile analysis results obtained by each laboratory for the CTX1B calibrant, segments represent the mean portions (*n* = 3) of 54-deoxyCTX1B (grey), 52-*epi*-54-deoxyCTX1B (white) and CTX1B (black); data obtained using standards with qNMR adjusted concentrations as well as the CTX3C calibrant are provided in the Supporting Information (Fig. S1, Fig. S2, Table S10); different letters for each CTX analogue and the total content represent a significant difference between the laboratories after pairwise comparison (One Way ANOVA, Tukey post hoc test, *p <* 0.01); y-axes of panel C and E are adjusted to the same dimension to enable direct comparison of the results obtained for the same matrix (fried fish fillet) and different extraction methods (C: Spielmeyer et al. (2021), E: Murray et al. (2018)); second y-axes showing the total content are adjusted to twice the scale of the corresponding first y-axes. (For interpretation of the references to colour in this figure legend, the reader is referred to the web version of this article.)

For samples 3 and 4, CTX1B contents determined by the five participants had (significantly) higher quantities reported by Lab A and D than by Labs B, C, and E, following the same trend as observed for sample 1 A which was prepared using the same procedure (Fig. 5 A, C, and Fig. 3). This indicates that the matrix effects observed for this extraction method (Spielmeyer et al., 2021), using different matrices (wet fish fillet, fish curry, fried fish), were consistent. A comparable trend was observed for the content of 52-*epi*-54-deoxyCTX1B, although the differences between Labs A/D and B/C/E were more pronounced (Fig. 5 A, C). For 54-deoxyCTX1B, the highest contents were determined by Lab A which were in the same range as the results from Labs B, C, and D. On the contrary, contents reported by Lab E were remarkably lower for this analogue, by factor 2.5 compared to the average of Labs A to D. This indicates different matrix effects for the individual CTX analogues, as determined for CTX1B and CTX3C within this study (Table S8), even though the analogues might elute adjacent to each other like 54-deoxyCTX1B and its 52-epimer. For the sum of all three analogues, the highest contents were determined by Labs A and D without significant difference between these two laboratories (Fig. 5 A, C). Compared to these two participants, Lab E reported a significantly lower total content for both samples (*p <* 0.01). Results reported by Labs B and C were in between these values, with no significant difference found between the two participants.

The reported contents for sample 5 followed different trends. Lab D reported the highest content for all three individual analogues (factor 2 higher than the average of the other participants) and consequently, the total content determined by Lab D was significantly higher than the total content of the other laboratories (*p <* 0.01) (Fig. 5 E, Table S10). Because sample 5 was the same fish as sample 4, but subjected to a different extraction and clean-up procedure, the matrix composition of this extract was likely different from the extract of sample 4. Different matrix compositions can result in different matrix effects, which in this case lead to a signal enhancement for Lab D and/or a signal suppression for the other participants. Results for Labs A, B, and C were in the same order of magnitude for all three analogues. As observed for samples 3 and 4, Lab E reported lower contents for 54-deoxyCTX1B and its 52-epimer and the lowest total content of all participants (Fig. 5 E). Surprisingly, the greatest difference was observed between Labs D and E, despite the fact that both used the same instrument manufacturer (different model), but however adopted different mobile phase compositions (acidic vs basic).

The highest and lowest total contents reported for each sample (i.e., samples 3, 4, and 5) differed by approximately a factor of 3. Thus, extracts obtained by both sample preparation protocols caused (variable) matrix effects, leading to significant differences in the quantitative results reported by the participants. In addition, the quality of the matrix effects differed, depending on the protocol used for sample preparation (and the setup used for analysis), which hampers the ability to compare results obtained for extracts prepared by different methods, both within one laboratory and between laboratories.

Samples 4 and 5 originated from the same matrix (fried fish) and extracts were prepared and cleaned-up in two different ways. For Labs B to E, CTX contents in sample 5 were higher than in sample 4 (factor 1.2 to 2.1) which indicates a loss of analyte(s) in the case of the sample preparation for sample 4, probably during SPE clean-up as recently reported (Loeffler & Spielmeyer, 2024). However, Lab A found lower CTX contents in sample 5 (factor 0.6 to 0.7) which would contradict this statement. As discussed for the matrix effects (Section 3.2), the determination and comparison of extraction efficiencies would be a more reliable parameter to evaluate sample preparation methods regarding analyte recovery. To date, no standardized CTX analyses protocol is available, and laboratories use individually customized sample preparation methods, potentially causing variations for the individual compounds and total CTX content in a sample. Universal extraction and clean-up methods are required that can sufficiently purify samples to reduce matrix effects and to improve comparability of results obtained on a variety of analytical instruments. Approaches like the development of certified reference material or interlaboratory validation frameworks would benefit from this advancement as well.

Depending on the laboratory, sample 4 showed a two to three times higher CTX content than sample 3 (Fig. 5 A and C, Table S10). Portions of sample 4 (fried fish) were initially used to prepare a fish curry (sample 3) and the consumption of the curry caused symptoms of ciguatera. The entire curry sample, including coconut milk and vegetables, was homogenized and used for further investigation (Murray et al., 2021). Thus, CTXs present in the fish were diluted by other meal ingredients, leading to a lower content in sample 3 compared to sample 4. Furthermore, processes such as frying and cooking can alter the water content of a sample. As CTX contents are calculated based on the sample weight used for extraction, water loss prior to sample preparation has an impact on the quantitative results. Freeze-drying of samples prior extraction could overcome this issue; however, not all sample preparation methods are optimized for the extraction of dried material. In this study, materials were not freeze-dried, but used as received. Another option for reporting CTX contents on dry weight basis would be the determination of the sample's water content using a separate tissue aliquot; however, such an approach might be hampered by the availability of sufficient material, especially for meal remnants. This aspect highlights another hurdle for the CTX research field, particularly when processed food is investigated or if guidance values should be established based on contents reported from outbreak samples (including analyses of meal remnants). Providing contents on a dry weight base might improve the comparability of the results being reported.

As the material of samples 3, 4, and 5 originated from the same fish fillet(s), similar profiles were expected to be present in the sample extracts. Comparable CTX profiles were obtained between laboratories and samples, although different extraction methods were used for the sample preparation and the CTX contents showed different trends in the respective samples (Fig. 5, Table S11). In all cases, 54-deoxyCTX1B was found to be the main congener, with a relative proportion of 43 to 67 % (calculated over all injections, *n* = 15). Whereas the proportion of its 52-epimer was 19 to 38 %. The total CTX contribution from 54-deoxyCTX1B and its 52-epimer ranged from 66 to 88 %. Here, a significant difference between Lab E and the other participants was found (*p <* 0.001). For Labs A to D, the sum of 54-deoxyCTX1B and its 52-epimer ranged from 81 to 88 %, whereas the proportion was 66 to 73 % for Lab E, which represents the (significantly) lower 54-deoxyCTX1B content determined by Lab E (Fig. 5). Consequently, the proportion of CTX1B ranged from 12 to 19 % for Labs A to D and from 27 to 34 % for Lab E (Fig. 5 B, D, F, Table S11).

Profiles of samples 3 and 4 were almost identical within one laboratory (Fig. 5 B, D). For sample 5, differences within the profile were observed for Labs B and C, with slightly varying proportions of 52-*epi*-54-deoxyCTX1B and 54-deoxyCTX1B, but not for the sum of the two analogues (Fig. 5 B, D, F, Table S11). Since Labs A, D, and E provided almost identical profiles for the three samples, an impact of the sample preparation itself (i.e., different extraction efficiencies for the individual analogues) appears unlikely. Matrix differences among samples in combination with the analytical conditions used by Labs B and C might cause profile shifts in these cases. Matrix effects for 54-deoxyCTX1B and its 52-epimer cannot be estimated from this study as the analytes were not available as standard compounds in sufficient quantities. Based on the results for samples 1 and 2, further sample clean-up (e.g., fractionation) should deliver a more uniform profile among all partners and samples.

A recent study has indicated a comparable potency for 54-deoxyCTX1B and its 52-epimer in the neuro-2a bioassay (Yokozeki et al., 2023). From that point of view, the sum of both analogues would be sufficient to estimate the toxicity of a sample. Profile analysis studies within one laboratory should deliver comparable results, if the same sample preparation method is used. When data are compared with results reported in the literature, it should be considered that minor differences in the profiles, as observed within this study, could be due to analytical issues only and not due to different ratios of the analogues in the individual samples.

#### 3.3.4 CTX3C group congeners (sample 6)

Sample 6 contained several analogues of the CTX3C group and, thus, a different profile than sample 2 (C-CTX-1) as well as samples 1 and 3 to 5 (CTX4A group analogues). Only peaks identified by at least one laboratory and by an external standard were included in the data analysis. Further details concerning compound identification are provided in Section 3.3.1. Based on these criteria, five analogues were considered for further analysis, namely CTX3C and its 49-epimer (CTX3B), 51-hydroxyCTX3C, and 2,3-dihydroxyCTX3C and its 49-epimer. CTX3C was not detected by Lab D, probably due to the low content in the sample. Several other peaks, potentially originating from mono- and trihydroxyCTX3C analogues were detected by the participants. Due to the lack of reference material(s), these compounds were not included in the quantitative evaluation. Lab A reported no peaks confirmed according to the HRMS criteria for the entire sample (see Section 3.3.1) which may be due to the comparable high LOD for CTX3C on the HRMS system (11 μg L^−1^). Thus, only the results from Labs B to E are presented in this section.

**Fig. 6:**
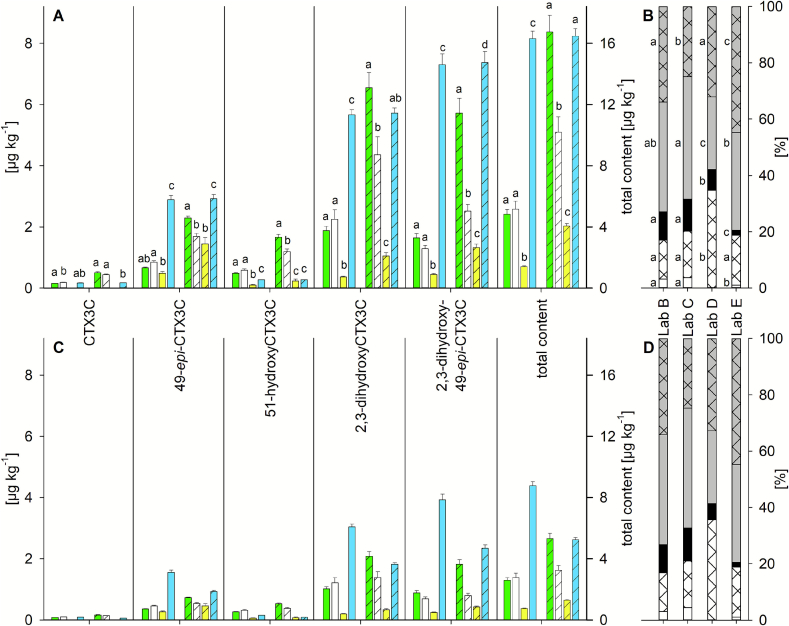
**(A,C)** Quantitative and **(B,D)** profile analysis results obtained for sample 6 for **(A,B)** the concentrations of the commercial standard and **(C,D)** the qNMR adjusted concentrations; bars in **(A,C)** represent the quantitative results obtained by (from left to right) Lab B (green), C (white), D (yellow), and E (blue) via the calibration of CTX1B (blank) and CTX3C (shaded); results are provided as mean, error bar shows standard deviation (*n* = 3); second y-axes showing the total content are adjusted to twice the scale of the corresponding first y-axes; different letters for each CTX analogue and the total content represent a significant difference between the laboratories for the respective calibrants after pairwise comparison (One Way ANOVA, Tukey post hoc test, *p <* 0.01); bars in **(B,D)** represent profile analysis results obtained by each laboratory using the **(B)** CTX1B and **(D)** CTX3C calibrant, segments represent portions of 2,3-dihydroxyCTX3C (grey) and its 49-epimer (grey shaded), 51-hydroxyCTX3C (black), CTX3C (white) and its 49-epimer (white shaded); for each CTX analogue, different letters represent a significant difference between the laboratories after pairwise comparison (One Way ANOVA, Tukey post hoc test, *p <* 0.01); for Lab A, no results were provided as no peaks were detected by this participant; for detailed data see also Table S12.

For the quantitative results, different trends were observed for the CTX3C group analogues compared to the analogues detected in samples 1 to 5. For example, the highest content was reported by Lab E in several cases, especially for the CTX1B calibrant (Fig. 6 A, C, Table S12). On the contrary, Lab D, who described some of the highest contents in samples 1 to 5, reported the lowest contents for sample 6 in all cases. Another major difference is the impact of the calibrant used for quantitation. For samples 1 to 5, results of the laboratories demonstrated good congruence for the CTX1B calibrant, whereas the CTX3C calibrant resulted in higher variability (Fig. 3, Fig. 4, Fig. 5). For sample 6, none of the calibrants led to uniform results among the participants and the results were significantly different (*p* < 0.01, Fig. 6 A, C). However, compared to CTX1B, the use of the CTX3C calibrant noticeably reduced the differences between the laboratories. The highest and lowest total contents differed by a factor of 11.6 and 4.1 for the CTX1B and CTX3C calibrants, respectively (Fig. 6 A, C, Table S12).

Contents of 49-*epi*-CTX3C and 51-hydroxyCTX3C determined by Lab D were comparable to contents determined by Labs B and C and Lab E, respectively. CTX3C contributed less than 5 % to the CTX profile, and the missing CTX3C detection in the case of Lab D had only a minor impact on its significantly lower total content. The higher discrepancies for the total content were mainly caused by the results of 2,3-dihydroxyCTX3C and its 49-epimer that contributed to over 50 % of the total CTX content (Fig. 6, Table S12, Table S13). Lab D determined a significantly lower content for these two analogues, suggesting a strong signal suppression for Lab D and/or a signal enhancement for the other participants.

Lab D showed potential signal enhancements for the CTX4A group analogues in samples 3 and 4 and for compounds eluting in a similar time range like the 2,3-dihydroxyCTX3C analogues in sample 6 (3.57 min and 3.23 min for 54-deoxyCTX1B and its 52-epimer compared to 3.33 and 3.00 min for 2,3-dihydroxyCTX3C and its 49-epimer, respectively). These differences in potential signal enhancement and suppression might be caused by the analytical method (e.g., eluent conditions, precursor ion selection), the matrix composition of the sample extract and/or the diverse CTX groups. Individual ion transitions were used by the laboratories which might experience different matrix effects as discussed for sample 2. The precursor ion used by Lab D for 2,3-dihydroxyCTX3C ([M+NH_4_]^+^) could be more susceptible to signal suppression than the precursor ions used by Lab E ([M+H]^+^) or Labs B and C ([M+Na]^+^) (Table S5) (see also discussion for sample 2, Section 3.3.2).

The samples 3, 4, and 6 were prepared according to the same protocol, but they represented different matrices (fish curry, fried fish, frozen wet fillet) and different species (*Plectropomus laevis*, *Lutjanus bohar*, see Table 1). The matrix constituents of the final extracts could be different, resulting in varying matrix effects and leading to the observed variations for the CTX4A and CTX3C group analogues. However, the results for samples 3 and 4 imply that the protocol used delivers at least comparable matrix effects over a certain range of matrices (Fig. 5 A, C) and the final extracts' composition of samples 3 and 4 is not expected to be orthogonal to sample 6. It should be considered that general differences between the two CTX groups concerning matrix effects exist, hypothesized to be caused by structural differences such as the side chain on ring A (Fig. 1). Consequently, method development and comparison studies should cover CTXs from several groups to get a comprehensive insight concerning the suitability of preparation and analytical methods to deliver congruent results.

For the profile, either 2,3-dihydroxyCTX3C (Labs B and C) or its 49-epimer (Labs D and E) were determined as the primary compound (Fig. 6 B, D, Table S13). The total CTX profile contribution from the sum of these two analogues ranged from 54 % to 80 % (calculated over all injections, *n* = 12). Lab D determined a significantly lower ratio for this sum (average of 59 % compared to 67 % to 80 %) and a significantly higher proportion for the sum of CTX3C and the 49-epimer than the other participants (36 % compared to 17 % to 21 %) (*p <* 0.01). These discrepancies are not the result of the missing CTX3C in the case of Lab D, but of the lower content determined for 2,3-dihydroxyCTX3C and its 49-epimer.

Labs B, C, and E showed no significant differences for the sum of CTX3C and its 49-epimer. For 51-hydroxyCTX3C, Lab E found a significantly lower (2 %) and Labs B and C a significantly higher proportion (10 % to 12 %) than Lab D (6 %). In total, CTX3C group profiles determined for sample 6 showed a higher variability among the participants than CTX4A group profiles determined for samples 3 to 5 (Fig. 6 B, D compared to Fig. 5 B, D, F, Table S11, Table S13). The highest similarity was observed for the profiles reported by Labs B and C. Both participants used the pseudo ion transition of the sodium adduct for quantitation and acidic eluent conditions. Labs D and E used instruments of the same manufacturer, but different eluents (acidic vs. basic conditions, see Table 2), emphasizing the potential impact of the eluent pH on the quantitation of CTXs. CTX3C group analogues (sample 6) appear to be more sensitive on the analytical setup (eluent conditions, ion transition used for quantitation) than analogues of the CTX4A group. For samples 3 to 5 (CTX4A group analogues), quantitative results differed significantly between the five laboratories, but the resulting profiles were almost identical, among different matrices and different extraction methods (Fig. 5, Table S11).

As discussed previously, an extended sample clean-up (e.g., fractionation) might reduce the observed discrepancies between the laboratories, and a total CTX content that better approximates a “true value” might be estimated. This could also help answer the question of which calibrant(s) would be optimal for the quantitation of CTX3C group analogues. So far, CTX3C appears to be more suitable than CTX1B. It should be noted that only one sample containing the CTX3C group profile was investigated in this study, by following a single sample preparation protocol. The aspect of defining suitable quantitation standards for CTX analogues, for which no reference material is available, should be investigated in detail in further studies.

### 3.4 Summary and future implications

Currently, there is no standardized protocol for CTX sample analysis and laboratories worldwide use their own analytical methods and instrumental setups. Therefore, to simulate the current status of CTX analysis by LC-MS/MS and LC-HRMS, no parameters were specified within this comparative study, apart from the CTX analogues that had to be included in the MS methods (Table S4). Through this flexible approach, several technical issues were identified by different laboratories that are critical to the implementation of CTX detection methods.

*Several solvents and eluent additives implemented in different laboratories were found suitable for analysis; however, conditions can lead to variable matrix effects.* – The eluents used by the participants and proven to be suitable were either methanol, acetonitrile, or a mixture of both (Table 2). Concerning the pH of the eluents, both acidic and basic conditions were shown to be suitable for the detection and quantitation of CTX analogues in different matrices. Even though the matrix effects of C-CTX-1 could not be properly investigated due to the absence of standard material, the sodium adduct of at least this analogue might be more prone to matrix effects under basic conditions, as observed in sample 2 (Fig. 4). Also, for analogues of the CTX3C group, results indicated a dependence of the matrix effects on the selected precursor ion (Fig. 6). These aspects should be investigated further, i.e., comparing different adducts under different eluent conditions in order to obtain a more comprehensive insight into the influence of these factors.

*Detection by HRMS adds further certainty to CTX analogue identification compared to low resolution LC-MS/MS methods, but methods might reveal higher detection limits.* – One participant used an HRMS (orbitrap) setup. Analyses by high resolution adds further certainty to the identification of CTX analogues that lack standards or reference material. However, one drawback is that these instruments often have a lower sensitivity (higher LOD/LOQ) than what can be obtained on triple quadrupole instruments of the same generation (Estevez et al., 2020; Sibat et al., 2018; Spielmeyer et al., 2021). As demonstrated herein, where no peaks were confirmed by the HRMS instrument in sample 6 but were detected by the triple quadrupole MS systems. Higher LODs could also be a result of non-optimal parameters for the CTX3C group analogues on the respective instrument. Such optimization is currently hindered by the lack of standards and reference material, which is essential for developing sensitive CTX detection methods.

*Method comparison studies need to include CTX analogues of several groups as analogues were influenced differently by analytical parameters.* – CTX contents followed different trends among laboratories and matrix effects appear to be dependent not only on the instruments and analytical parameters used by the participants, but also on the CTX group. As shown for the six samples investigated herein, C-CTX-1 and analogues of the CTX4A and CTX3C groups can respond independently to the same variable, e. g., the suitable calibrant. The results underline the necessity to include CTX analogues of all CTX groups (where available) in such comparison studies, as a strict focus on a single CTX group would limit the applicability, resulting in the understanding of only the CTX analyte (and group) tested. In this study, no mixed profiles were investigated and CTX groups were recovered from different naturally incurred matrices. Materials containing a mixed profile (e.g., CTX4A and CTX3C group analogues) would be the optimum for covering as many analytical aspects as possible within one sample and analysis. For instance, the applicability of CTX1B and CTX3C as calibrants for analogues of different CTX groups could be directly compared within one sample.

*For development (and potential harmonization) of sample preparation methods, the final extracts obtained should be analyzed on different instruments.* – According to the matrix effect study results and samples 1 and 2, the best option for the development of a sample preparation protocol would be to test the final extract on several analytical instruments. With this approach, matrix effects could be evaluated under exhaustive conditions (e.g., different instruments, eluents, and source parameters). As shown for samples 1 and 2, quantitative results aligned well for the fractionated samples (potentially higher extract purity), but for extracts with lower purity both signal enhancement and signal suppression were observed for the same sample analyzed using different setups. Analyses on different instruments could provide a good indication of the extract purity and the effect of remaining matrix constituents on the quantitation. In addition, the extraction efficiency of a sample preparation method should be determined, as this parameter allows for an evaluation of analyte recovery, independent of the matrix effect on the instrument and the conditions used for analysis. Using this parameter, sample preparation protocols could be compared, and potential advantages and problems could be better identified for the respective protocols. This can also support the development and validation of standard operating protocols. The comparison of different sample extraction methods was not the focus of this study, thus, no evaluation of different recovery rates or extraction efficiencies was performed. The two different protocols applied in this study (fried fish, samples 4 and 5) led to variable matrix effects for each participant and, consequently, to different quantitative outcomes. This observation should be considered if quantitative CTX results reported in literature are evaluated or compared with results obtained by one's own method.

*Quantitation should be performed with CTX analogues belonging to the same CTX group like the respective analyte.* – CTX1B and CTX3C standards showed different responses for all analytical setups. For acidic eluent conditions, CTX3C showed a lower response than CTX1B in all cases whereas basic conditions resulted in comparable responses (commercial standard concentrations) or a higher response of CTX3C (qNMR adjusted concentrations). The results of this study imply that a CTX analogue belonging to the same group should be used as a reference for quantitation, if standards for each individual analogue are not available (e. g., CTX1B for the CTX4A group, CTX3C for the CTX3C group). If no analogue for the respective group is available (like C-CTX-1), the best approach might be to choose a reference as similar as possible to the respective analyte. Within this study, results for C-CTX-1 in sample 2 (Fig. 4) were more congruent using the CTX1B calibrant.

The development of internal standards suitable for CTX analysis (e. g., isotopic labelled compounds) would help advance the field of CTX quantitation by LC-MS based methods. However, it should be noted that commercially available CTX standards are not synthetic compounds, but rather purified from algae or fish. These materials are currently not certified and no information regarding their purity was provided by the manufacturer. This adds some uncertainty as suggested by the comparison of the commercially available standards with those quantified by qNMR. Thus, details concerning compound purity (best case: qNMR data) should be provided by the manufacturer(s). LOT numbers of the standards used within a study should be described by the researchers to enhance comparability of results reported by different groups.

*Future outlook* – When certified standards coupled with standardized extraction and analytical protocols are available, another aspect of quantitative analysis might become relevant. CTXs show different potencies in cell-based assays, resulting in different toxicity equivalent factors (TEFs) (Yokozeki et al., 2023). For paralytic shellfish poisoning (PSP) toxins, the TEFs are used to calculate the respective toxin contents in saxitoxin equivalents diHCl per kilogram (Commission Implementing Regulation (EU) 2019/627). Current EU legislation does not define a limit value for CTXs, but each positive CTX detection leads to a ban (or withdrawal) from the market, making this point a gratuitous discussion. Considering monitoring programs for the occurrence of CTXs and toxicological studies (both relevant for risk assessments of CTXs in seafood or proper management measures by competent authorities) or the development of medical treatments of CP, accurate CTX analysis can be relevant, apart from legislative aspects. Therefore, a focused effort should be made to improve the comparability and congruence of CTX analyses results through exhaustive method comparison studies.

## CRediT authorship contribution statement

**Astrid Spielmeyer:** Writing – review & editing, Writing – original draft, Visualization, Resources, Investigation, Conceptualization. **Vincent Hort:** Writing – review & editing, Writing – original draft, Visualization, Investigation, Conceptualization. **J. Sam Murray:** Writing – review & editing, Writing – original draft, Visualization, Resources, Investigation, Conceptualization. **Cintia Flores:** Writing – review & editing, Writing – original draft, Visualization, Investigation, Conceptualization. **Andres Sanchez-Henao:** Writing – review & editing, Investigation. **Emillie M.F. Passfield:** Investigation. **Caroline Desbourdes:** Investigation. **Lourdes Barreiro-Crespo:** Investigation. **Mònica Campàs:** Writing – review & editing, Conceptualization. **Jorge Diogène:** Writing – review & editing, Conceptualization. **Fernando Real Valcárcel:** Resources. **Jean Turquet:** Resources. **Christopher R. Loeffler:** Writing – review & editing, Resources, Investigation, Conceptualization. **Maria Rambla-Alegre:** Writing – review & editing, Writing – original draft, Visualization, Resources, Investigation, Conceptualization.

## Declaration of competing interest

The authors declare that they have no known competing financial interests or personal relationships that could have appeared to influence the work reported in this paper.

## Data Availability

Data will be made available on request.

## Appendix A Supplementary data

Supplementary material Image 1
