# Supplementary material for: Comparison of LC-MS methods for the quantitation of ciguatoxins in fish – A collaborative study
Source: Food Chem X. 2025 Nov 9;32:103277. doi: 10.1016/j.fochx.2025.103277 (PMC12663502; doi:10.1016/j.fochx.2025.103277)
Supplement: Supplementary file 1 — Supplementary material [file mmc1.pdf]

Supporting Information

Comparison of LC-MS methods for the quantitation of ciguatoxins – A collaborative study

Astrid Spielmeyer[a], Vincent Hort[b], J. Sam Murray[c], Cintia Flores[d], Andres Sanchez-Henao[e,f], Emillie M. F. Passfield[c], Caroline Desbourdes[b], Lourdes Barreiro-Crespo[e,g], Mònica Campàs[e], Jorge Diogène[e], Fernando Real Valcárcel[f], Jean Turquet[h], Christopher R. Loeffler[a], Maria Rambla-Alegre[e]

This Supporting information contains 13 Tables and 2 Figures.

Content:

Table S1. Pipetting scheme for preparation of calibration standards.

Table S2. Peak areas for the comparison of the commercial standards and standards checked by qNMR.

Table S3. Sequence used for analysis.

Table S4. Accurate *m/z* signals of the CTX congeners investigated in this study.

Table S5. (Pseudo) ion transitions respective extraction masses utilized by each laboratory.

Table S6. Data of calibration functions obtained for CTX1B and CTX3C in matrix-free standard solutions.

Table S7. Measurement precision of calibration standards depending on CTX analogue and concentration.

Table S8. Matrix effects determined for CTX1B and CTX3C added to an extract of *Caranx* spp.

Table S9. Contents of CTX1B (samples 1) and C-CTX-1 (samples 2) determined by each participant.

Table S10. Contents of 54-deoxyCTX1B, 52-*epi*-54-deoxyCTX1B, and CTX1B as well as the total content in samples 3, 4, and 5 determined by each participant.

Table S11. Mean proportion of the analogues and ratio of 54-deoxyCTX1B and its 52-epimer detected in samples 3 to 5.

Table S12. Contents of CTX3C group analogues determined in sample 6 by each participant.

Table S13. Mean proportion of the analogues detected in samples 6.

Figure S1. Quantitative and profile analysis results obtained samples 3 to 5.

Figure S2. Quantitative and profile analysis results obtained samples 3 to 5 (concentration adjusted by qNMR standard).

Table S1. Pipetting scheme for preparation of calibration standards.

CTX1B[1] [µL]	CTX3C[1] [µL]	methanol [µL]		final concentration [µg L^{-1}]
50	50	0		10.0
37.5	37.5	25		7.5
50	50	100		5.0
35	35	130		3.5
20	20	160		2.0
			mixed standard[2] [µL]	
		50	50	1.0
		150	50	0.5

[1] 20 µg L^{-1}; [2] 2 µg L^{-1}

Table S2. Peak areas for the comparison of the commercial standards and standards checked by qNMR.

	CTX1B		CTX3C	
	commercial	qNMR	commercial	qNMR
injection 1	33056	61304	7182	23383
injection 2	34186	62961	7600	22988
injection 3	33581	63400	7285	23079
mean ± SD	33608 ± 565	62555 ± 1105	7356 ± 218	23150 ± 207
concentration determined by qNMR*		10		10
calculated concentration of commercial standards	5.4		3.2	

Concentrations provided in µg L^{-1}, SD – standard deviation, * for details see Kato and Yasumoto (2017), standards were kindly provided by Prof. Yasumoto (Japan Food Research Laboratories JFRL, Tokyo, Japan).

Table S3. Sequence used for analysis.

matrix effect, standard #1
matrix effect, matrix sample #1
matrix effect, standard #2
matrix effect, matrix sample #2
matrix effect, standard #3
matrix effect, matrix sample #3
methanol
calibration standards 0.5 to 10 µg L^{-1} #1
methanol
sample set #1:
sample 6 #1
sample 1 (A, B, C) #1
sample 2 (A, B, C) #1
sample 5 #1
sample 3 #1
sample 4 #1
methanol
calibration standards 0.5 to 10 µg L^{-1} #2
methanol
sample set #2
methanol
calibration standards 0.5 to 10 µg L^{-1} #3
methanol
sample set #3
methanol

Table S4. Accurate *m/z* signals of the CTX congeners investigated in this study.

CTX group	CTX congener	also covers	Formula	$[M+H-2H_2O]^+$	$[M+H-H_2O]^+$	$[M+H]^+$	$[M+NH_4]^+$	$[M+Na]^+$
CTX4A	CTX4A	52-*epi*-CTX4A	$C_{60}H_{84}O_{16}$	1025.56208	1043.57265	1061.58321	1078.60976	1083.56516
	M-*seco*-CTX4A	52-*epi*-M-*seco*-CTX4A	$C_{60}H_{86}O_{17}$	1043.57265	1061.58321	1079.59378	1096.62033	1101.57572
	54-deoxyCTX1B	52-*epi*-54-deoxyCTX1B	$C_{60}H_{86}O_{18}$	1059.56756	1077.57813	1095.58869	1112.61524	1117.57064
	CTX1B	52-*epi*-CTX1B, 54-*epi*-CTX1B, 52-*epi*-54-*epi*-CTX1B, 54-deoxy-50-hydroxyCTX1B	$C_{60}H_{86}O_{19}$	1075.56248	1093.57304	1111.58361	1128.61016	1133.56555
CTX3C	CTX3C	49-*epi*-CTX3C	$C_{57}H_{82}O_{16}$	987.54643	1005.55700	1023.56756	1040.59411	1045.54951
	51-hydroxyCTX3C		$C_{57}H_{82}O_{17}$	1003.54135	1021.55191	1039.56248	1056.58903	1061.54442
	M-*seco*-CTX3C	49-*epi*-M-*seco*-CTX3C, 2-hydroxyCTX3C, 49-*epi*-2-hydroxyCTX3C	$C_{57}H_{84}O_{17}$	1005.55700	1023.56756	1041.57813	1058.60468	1063.56007
	51-hydroxy-2-oxoCTX3C		$C_{57}H_{82}O_{18}$	1019.53626	1037.54683	1055.55739	1072.58394	1077.53934
	2,3-dihydroxyCTX3C	49-*epi*-2,3-dihydroxyCTX3C	$C_{57}H_{84}O_{18}$	1021.55191	1039.56248	1057.57304	1074.59959	1079.55499
	A-*seco*-51-hydroxyCTX3C		$C_{57}H_{86}O_{18}$	1023.56756	1041.57813	1059.58869	1076.61524	1081.57064
	2,3,51-trihydroxyCTX3C		$C_{57}H_{84}O_{19}$	1037.54683	1055.55739	1073.56796	1090.59451	1095.54990
C-/I-CTX	C-CTX-1	56-*epi*-C-CTX-1, I-CTX-1, I-CTX-2	$C_{62}H_{92}O_{19}$	1105.60943	1123.61999	1141.63056	1158.65711	1163.61250
	C-CTX-3	C-CTX-4	$C_{62}H_{94}O_{19}$	1107.62508	1125.63564	1143.64621	1160.67276	1165.62815
	C-CTX-5	I-CTX-5	$C_{62}H_{90}O_{19}$	1103.59378	1121.60434	1139.61491	1156.64146	1161.59685

Table S5. (Pseudo) ion transitions respective extraction masses utilized by each laboratory.

congener	Laboratory A	Laboratory B (precursor / product ion)	Laboratory C (precursor / product ion)	Laboratory D (precursor / product ion)	Laboratory E (precursor / product ions)
CTX4A[1]				$[M+H]^+$ / 125.1; 155.1	$[M+H]^+$ / 125.1; 155.1
M-*seco*-CTX4A[1]				$[M+NH_4]^+$ / $[M+H-H_2O]^+$; 125.1	$[M+H]^+$ / 143.1; 173.1
54-deoxyCTX1B[1]				$[M+NH_4]^+$ / **$[M+H-H_2O]^+$**; $[M+H-2H_2O]^+$	$[M+NH_4]^+$ / 1041.8; **$[M+H-H_2O]^+$**<br>$[M+Na]^+$ / $[M+Na]^+$
CTX1B[1]				$[M+NH_4]^+$ / **95.0**; 109.0	$[M+NH_4]^+$ / **95.0**; 109.0<br>$[M+Na]^+$ / $[M+Na]^+$
CTX3C[2]				$[M+H]^+$ / **125.1**; 155.1	$[M+H]^+$ / **125.1**; 155.1
51-hydroxyCTX3C				$[M+NH_4]^+$ / $[M+H-H_2O]^+$; **95.0**	$[M+H]^+$ / 141.1; 171.1<br>**$[M+Na]^+$ / $[M+Na]^+$**
2-hydroxyCTX3C[2]				$[M+H-H_2O]^+$ / $[M+H-2H_2O]^+$; 125.1	$[M+H]^+$ / 125.1; 155.1
M-*seco*-CTX3C[2]				$[M+H-H_2O]^+$ / $[M+H-2H_2O]^+$; 125.1	$[M+H]^+$ / 143.1; 173.1
51-hydroxy-2-oxoCTX3C				$[M+NH_4]^+$ / $[M+H-H_2O]^+$; $[M+H-2H_2O]^+$	$[M+H]^+$ / 141.1; 171.1<br>$[M+Na]^+$ / $[M+Na]^+$
2,3-dihydroxyCTX3C[2]				$[M+NH_4]^+$ / **$[M+H-H_2O]^+$**; $[M+H-2H_2O]^+$	$[M+H]^+$ / **125.1**; 155.1<br>$[M+Na]^+$ / $[M+Na]^+$
A-*seco*-51-hydroxyCTX3C				$[M+NH_4]^+$ / $[M+H-H_2O]^+$; $[M+H-2H_2O]^+$	$[M+H]^+$ / 141.1; 171.1<br>$[M+Na]^+$ / $[M+Na]^+$
2,3,51-trihydroxyCTX3C				$[M+NH_4]^+$ / $[M+H-H_2O]^+$; $[M+H-2H_2O]^+$	$[M+H]^+$ / 141.1<br>$[M+Na]^+$ / $[M+Na]^+$
C-CTX-3[3]				$[M+H]^+$ / $[M+H-H_2O]^+$; 108.9	$[M+H]^+$ / $[M+H-H_2O]^+$<br>$[M+NH_4]^+$ / $[M+H-H_2O]^+$
C-CTX-5				$[M+NH_4]^+$ / $[M+H]^+$; 108.9	$[M+H]^+$ / $[M+H-H_2O]^+$<br>$[M+NH_4]^+$ / $[M+H-H_2O]^+$
C-CTX-1[3]				$[M+NH_4]^+$ / **$[M+H-H_2O]^+$**; 191.1	**$[M+H-H_2O]^+$ / $[M+H-3H_2O]^+$**<br>$[M+Na]^+$ / $[M+Na]^+$
All congeners	Extraction of **$[M+Na]^+$, $[M+NH_4]^+$, $[M+H]^+$** from Full Scan (accurate mass window: 10 ppm)	**$[M+Na]^+$ / $[M+Na]^+$**	**$[M+Na]^+$ / $[M+Na]^+$**		

Additional parameters	Capillary voltage: 47.5 V<br>Tube lens voltage: 186 V<br>Skimmer voltage: 18 V<br>Scan rate: 2 Hz	Source fragmentation: 40 V<br>RF Lens: 250 V<br>Collision Energy: 45 eV<br>Cycle time: 600 msec	Declustering potential: 80 V<br>Entrance potential: 6 V<br>Cell Exit potential: 18 V<br>Collision energy: 60 eV<br>Dwell time: 50 msec	Cone potential: 20 V<br>Collision energy: 20 eV for loss of water / ammonia;<br>80 eV for CTX-1B, rest 70 eV<br>Dwell time: 14 msec	Cone voltage: 30 V<br>Collision energy: 15/50 eV for C-CTXs; 55/65 eV for CTX-1B, 20/25/55 eV for 54-deoxyCTX-1B, rest 50 eV<br>Dwell time: 50 msec for C-CTXs, rest 10 msec

[1] Same conditions for the 52- and 54-epimer; [2] same conditions for the 49-epimer; [3] same conditions for the 56-epimer; the corresponding *m/z* values of the ions are provided in Table S3; ion transitions highlighted in bold were used for quantitation of CTX analogues detected in the study samples.

Table S6. Data of calibration functions obtained for CTX1B and CTX3C in matrix-free standard solutions.

	retention time [min]		measurement precision [%]		correlation coefficient R^2		slope		relative slope[a]	slope (qNMR corrected concentrations)		relative slope[b]
	CTX1B	CTX3C	CTX1B	CTX3C	CTX1B	CTX3C	CTX1B	CTX3C	CTX3C/CTX1B	CTX1B	CTX3C	CTX3C/CTX1B
Lab A[c]	3.51	11.44	1.0 - 14.2		0.990		17027			31694		
Lab B	2.50	8.83	4.3 - 23.4	1.9 - 23.0	0.978	0.984	21229	6060	0.29	39514	19072	0.48
Lab C	3.28	9.79	5.1 - 11.3	9.9 - 19.3	0.985	0.975	248117	130770	0.53	461829	411564	0.89
Lab D	2.28	4.85	1.8 - 11.0	4.2 - 9.4	0.993	0.987	334	105	0.31	622	331	0.53
Lab E	3.94	6.76	6.4 - 33.2	2.7 - 21.2	0.941	0.978	842	834	0.99	1567	2624	1.67

[a] Ratio of the slope obtained for the CTX3C calibration function to that of the CTX1B calibration function based on the commercial standard concentrations; [b] ratio of the slope obtained for the CTX3C calibration function to that of the CTX1B calibration function based on the qNMR corrected concentrations (details provided in Section 2.1); [c] no data available for CTX3C as concentrations were <LOD (11 µg L^{-1}).

Table S7. Measurement precision of calibration standards depending on CTX analogue and concentration.

	0.5 µg L^{-1}	1.0 µg L^{-1}	2.0 µg L^{-1}	3.5 µg L^{-1}	5.0 µg L^{-1}	7.5 µg L^{-1}	10 µg L^{-1}
Lab				CTX1B			
A	7.0	14.2	5.9	2.4	1.0	2.1	1.2
B	9.5	9.5	8.6	15.1	23.4	10.5	4.3
C	11.3	9.6	6.7	5.1	10.3	9.6	7.6
D	7.8	11.0	6.8	6.3	1.8	4.3	5.3
E	33.2	6.4	15.6	20.6	18.3	13.0	13.1
				CTX3C			
B	12.7	6.4	23.0	1.9	5.1	9.3	9.0
C	19.3	16.6	9.9	13.1	13.8	12.0	12.0
D	6.8	7.7	4.2	8.9	5.9*	9.4	4.7
E	21.2	2.7	8.1	7.9	6.8	6.6	5.6

precision data provided in %, * n = 2

Table S8. Matrix effects determined for CTX1B and CTX3C added to an extract of *Caranx* spp.

	Matrix effect*	
	CTX1B	CTX3C
Lab A	+8%	**
Lab B	-26%	+115%
Lab C	-32%	-10%
Lab D	+5%	-25%
Lab E	-23%	-72%

* Calculated according to Equation (1) (Section 2.3 in the main article); ** no value provided as concentration < LOD.

Table S9. Contents of CTX1B (samples 1 A-C) and C-CTX-1 (samples 2 A-C) determined by each participant.

Sample	Lab	calibrant			
		CTX1B (commercial)	CTX3C (commercial)	CTX1B (qNMR adjusted)	CTX3C (qNMR adjusted)
1A	A	0.613 ± 0.030	NA	0.329 ± 0.016	NA
	B	0.117 ± 0.011	0.390 ± 0.039	0.063 ± 0.006	0.124 ± 0.012
	C	0.096 ± 0.006	0.222 ± 0.012	0.052 ± 0.003	0.071 ± 0.004
	D	0.476 ± 0.019	1.464 ± 0.061	0.256 ± 0.010	0.465 ± 0.019
	E	0.211 ± 0.021	0.213 ± 0.021	0.113 ± 0.011	0.068 ± 0.007
	all	0.303 ± 0.214	0.572 ± 0.544	0.163 ± 0.115	0.182 ± 0.173
1B	A	0.575 ± 0.014	NA	0.309 ± 0.008	NA
	B	0.165 ± 0.020	0.530 ± 0.071	0.089 ± 0.011	0.168 ± 0.022
	C	0.169 ± 0.009	0.421 ± 0.016	0.091 ± 0.005	0.134 ± 0.005
	D	0.401 ± 0.038	1.151 ± 0.119	0.215 ± 0.020	0.366 ± 0.038
	E	0.318 ± 0.024	0.323 ± 0.024	0.171 ± 0.013	0.103 ± 0.008
	all	0.326 ± 0.160	0.606 ± 0.343	0.175 ± 0.086	0.193 ± 0.109
1C	A	0.311 ± 0.006	NA	0.167 ± 0.003	NA
	B	0.240 ± 0.033	0.801 ± 0.114	0.129 ± 0.017	0.254 ± 0.036
	C	0.334 ± 0.014	0.714 ± 0.026	0.179 ± 0.007	0.227 ± 0.008
	D	0.308 ± 0.054	0.882 ± 0.172	0.166 ± 0.029	0.280 ± 0.055
	E	0.349 ± 0.046	0.353 ± 0.046	0.187 ± 0.024	0.112 ± 0.015
	all	0.308 ± 0.049	0.687 ± 0.230	0.166 ± 0.026	0.218 ± 0.073
2A	A	0.178 ± 0.019	NA	0.096 ± 0.010	NA
	B	0.141 ± 0.014	0.474 ± 0.050	0.076 ± 0.008	0.151 ± 0.016
	C	0.109 ± 0.014	0.247 ± 0.026	0.059 ± 0.007	0.078 ± 0.008
	D	0.366 ± 0.017	1.116 ± 0.054	0.197 ± 0.009	0.355 ± 0.017
	E	0.108 ± 0.010	0.109 ± 0.010	0.058 ± 0.005	0.035 ± 0.003
	all	0.180 ± 0.101	0.486 ± 0.405	0.097 ± 0.054	0.155 ± 0.129
2B	A	0.137 ± 0.016	NA	0.074 ± 0.009	NA
	B	0.154 ± 0.010	0.490 ± 0.035	0.083 ± 0.005	0.156 ± 0.011
	C	0.109 ± 0.043	0.306 ± 0.082	0.058 ± 0.023	0.097 ± 0.026
	D	0.266 ± 0.043	0.724 ± 0.138	0.143 ± 0.023	0.230 ± 0.044
	E	0.171 ± 0.002	0.174 ± 0.002	0.092 ± 0.001	0.055 ± 0.001
	all	0.167 ± 0.060	0.423 ± 0.227	0.090 ± 0.032	0.135 ± 0.072
2C	A	0.127 ± 0.015	NA	0.068 ± 0.008	NA
	B	0.110 ± 0.018	0.346 ± 0.062	0.059 ± 0.009	0.110 ± 0.020
	C	0.188 ± 0.015	0.437 ± 0.028	0.101 ± 0.008	0.139 ± 0.009
	D	0.216 ± 0.029	0.589 ± 0.092	0.116 ± 0.016	0.187 ± 0.029
	E	0.144 ± 0.012	0.147 ± 0.012	0.077 ± 0.006	0.047 ± 0.004
	all	0.157 ± 0.044	0.380 ± 0.174	0.084 ± 0.023	0.121 ± 0.055

Contents provided in µg kg^{-1} as mean ± SD for each lab (n=3) and for all labs combined (n=15 or n=12); three decimal places are provided for higher comprehensiveness of the data; contents for Lab E and sample 2 refer to the ion transition $[M+H-H_2O]^+$ / $[M+H-3H_2O]^+$; NA – data not available as CTX3C calibration levels were below the LOD of the system.

Table S10. Contents of 54-deoxyCTX1B, 52-*epi*-54-deoxyCTX1B, and CTX1B as well as the total content in samples 3, 4, and 5 determined by each participant.

Sample	Lab	calibrant			
		CTX1B (commercial)	CTX3C (commercial)	CTX1B (qNMR adjusted)	CTX3C (qNMR adjusted)
54-deoxyCTX1B					
3	A	0.873 ± 0.008	NA	0.469 ± 0.004	NA
	B	0.568 ± 0.099	1.971 ± 0.345	0.305 ± 0.053	0.626 ± 0.110
	C	0.522 ± 0.041	1.030 ± 0.077	0.280 ± 0.022	0.327 ± 0.025
	D	0.745 ± 0.046	2.321 ± 0.148	0.400 ± 0.025	0.737 ± 0.047
	E	0.261 ± 0.026	0.264 ± 0.026	0.140 ± 0.014	0.084 ± 0.008
	all	0.594 ± 0.221	1.396 ± 0.858	0.319 ± 0.119	0.444 ± 0.273
4	A	2.095 ± 0.250	NA	1.126 ± 0.135	NA
	B	1.518 ± 0.068	5.269 ± 0.240	0.816 ± 0.037	1.674 ± 0.076
	C	1.261 ± 0.311	2.493 ± 0.590	0.678 ± 0.167	0.792 ± 0.187
	D	1.566 ± 0.038	4.857 ± 0.122	0.842 ± 0.021	1.543 ± 0.039
	E	0.634 ± 0.037	0.642 ± 0.037	0.341 ± 0.020	0.204 ± 0.012
	all	1.415 ± 0.516	3.315 ± 1.975	0.760 ± 0.277	1.053 ± 0.627
5	A	1.449 ± 0.020	NA	0.778 ± 0.011	NA
	B	1.818 ± 0.168	6.302 ± 0.590	0.977 ± 0.091	2.002 ± 0.188
	C	1.535 ± 0.096	3.046 ± 0.183	0.825 ± 0.052	0.968 ± 0.058
	D	2.972 ± 0.050	9.283 ± 0.159	1.596 ± 0.027	2.950 ± 0.050
	E	0.889 ± 0.098	0.900 ± 0.099	0.478 ± 0.053	0.286 ± 0.031
	all	1.733 ± 0.718	4.883 ± 3.340	0.931 ± 0.386	1.551 ± 1.061
52-*epi*-54-deoxyCTX1B					
3	A	0.537 ± 0.031	NA	0.289 ± 0.017	NA
	B	0.172 ± 0.025	0.582 ± 0.089	0.092 ± 0.014	0.185 ± 0.028
	C	0.240 ± 0.027	0.495 ± 0.051	0.129 ± 0.014	0.157 ± 0.016
	D	0.550 ± 0.012	1.699 ± 0.037	0.295 ± 0.006	0.540 ± 0.012
	E	0.129 ± 0.006	0.131 ± 0.006	0.069 ± 0.003	0.041 ± 0.002
	all	0.326 ± 0.189	0.727 ± 0.614	0.175 ± 0.101	0.231 ± 0.195
4	A	1.262 ± 0.172	NA	0.678 ± 0.092	NA
	B	0.467 ± 0.016	1.585 ± 0.056	0.251 ± 0.009	0.504 ± 0.018
	C	0.613 ± 0.156	1.264 ± 0.296	0.330 ± 0.084	0.402 ± 0.094
	D	1.147 ± 0.023	3.524 ± 0.072	0.616 ± 0.012	1.120 ± 0.023
	E	0.332 ± 0.041	0.337 ± 0.041	0.178 ± 0.022	0.107 ± 0.013
	all	0.764 ± 0.395	1.677 ± 1.219	0.411 ± 0.212	0.533 ± 0.387
5	A	0.699 ± 0.009	NA	0.376 ± 0.005	NA
	B	0.887 ± 0.073	3.043 ± 0.255	0.477 ± 0.039	0.967 ± 0.081
	C	1.289 ± 0.130	2.579 ± 0.246	0.693 ± 0.070	0.819 ± 0.078
	D	2.374 ± 0.021	7.384 ± 0.066	1.275 ± 0.011	2.346 ± 0.021
	E	0.463 ± 0.073	0.469 ± 0.074	0.249 ± 0.039	0.149 ± 0.023
	all	1.143 ± 0.699	3.369 ± 2.629	0.614 ± 0.376	1.070 ± 0.835
CTX1B					
3	A	0.257 ± 0.004	NA	0.138 ± 0.002	NA
	B	0.125 ± 0.019	0.418 ± 0.066	0.067 ± 0.010	0.133 ± 0.021
	C	0.134 ± 0.014	0.295 ± 0.027	0.072 ± 0.008	0.094 ± 0.008
	D	0.237 ± 0.012	0.705 ± 0.037	0.127 ± 0.006	0.224 ± 0.012
	E	0.152 ± 0.008	0.154 ± 0.008	0.082 ± 0.004	0.049 ± 0.003
	all	0.181 ± 0.058	0.393 ± 0.215	0.097 ± 0.031	0.125 ± 0.068
4	A	0.669 ± 0.084	NA	0.360 ± 0.045	NA
	B	0.388 ± 0.066	1.311 ± 0.233	0.209 ± 0.036	0.417 ± 0.074
	C	0.385 ± 0.105	0.830 ± 0.199	0.207 ± 0.056	0.264 ± 0.063

	D	0.580 ± 0.016	1.722 ± 0.050	0.312 ± 0.008	0.547 ± 0.016
	E	0.400 ± 0.039	0.405 ± 0.040	0.215 ± 0.021	0.129 ± 0.013
	all	0.485 ± 0.136	1.067 ± 0.535	0.260 ± 0.073	0.339 ± 0.170
	A	0.451 ± 0.038	NA	0.242 ± 0.020	NA
	B	0.546 ± 0.024	1.846 ± 0.083	0.293 ± 0.013	0.586 ± 0.026
5	C	0.653 ± 0.054	1.372 ± 0.103	0.351 ± 0.029	0.436 ± 0.033
	D	1.155 ± 0.025	3.508 ± 0.081	0.620 ± 0.014	1.115 ± 0.026
	E	0.615 ± 0.044	0.622 ± 0.044	0.330 ± 0.024	0.198 ± 0.014
	all	0.684 ± 0.256	1.837 ± 1.108	0.367 ± 0.138	0.584 ± 0.352
total content					
	A	1.668 ± 0.030	NA	0.896 ± 0.016	NA
	B	0.865 ± 0.126	2.971 ± 0.442	0.465 ± 0.068	0.944 ± 0.141
3	C	0.896 ± 0.080	1.820 ± 0.151	0.481 ± 0.043	0.578 ± 0.048
	D	1.532 ± 0.060	4.725 ± 0.191	0.823 ± 0.032	1.501 ± 0.061
	E	0.541 ± 0.035	0.548 ± 0.036	0.291 ± 0.019	0.174 ± 0.011
	all	1.101 ± 0.448	2.516 ± 1.619	0.591 ± 0.241	0.799 ± 0.514
	A	4.026 ± 0.500	NA	2.163 ± 0.269	NA
	B	2.373 ± 0.101	8.166 ± 0.355	1.275 ± 0.054	2.595 ± 0.113
4	C	2.260 ± 0.569	4.586 ± 1.080	1.214 ± 0.306	1.457 ± 0.343
	D	3.294 ± 0.045	10.103 ± 0.143	1.770 ± 0.024	3.210 ± 0.045
	E	1.366 ± 0.061	1.383 ± 0.061	0.734 ± 0.033	0.440 ± 0.019
	all	2.664 ± 0.990	6.060 ± 3.530	1.431 ± 0.532	1.925 ± 1.122
	A	2.599 ± 0.040	NA	1.396 ± 0.021	NA
	B	3.251 ± 0.263	11.191 ± 0.920	1.747 ± 0.141	3.556 ± 0.292
5	C	3.477 ± 0.274	6.996 ± 0.520	1.868 ± 0.147	2.223 ± 0.165
	D	6.500 ± 0.046	20.175 ± 0.148	3.492 ± 0.025	6.410 ± 0.047
	E	1.967 ± 0.182	1.992 ± 0.184	1.057 ± 0.098	0.633 ± 0.058
	all	3.559 ± 1.626	10.088 ± 6.984	1.912 ± 0.873	3.205 ± 2.219

Contents provided in µg kg^{-1} as mean ± SD for each lab (n=3) and for all labs combined (n=15 or n=12); three decimal places are provided for higher comprehensiveness of the data; NA – data not available as CTX3C calibration levels were below the LOD of the system.

Table S11. Mean proportion of the analogues and ratio of 54-deoxyCTX1B and its 52-epimer detected in samples 3 to 5.

sample		A	B	C	D	E
3	CTX1B	15%	15%	15%	15%	28%
	52-*epi*-54-deoxyCTX1B	52%	66%	58%	49%	48%
	54-deoxyCTX1B	32%	20%	27%	36%	24%
	sum of 54-deoxyCTX1B and 52-epi-54deoxyCTX1B	85%	85%	85%	85%	72%
	ratio 54-deoxyCTX1B / 52-*epi*-54-deoxyCTX1B	1.6	3.3	2.2	1.4	2.0
4	CTX1B	17%	16%	17%	18%	29%
	52-*epi*-54-deoxyCTX1B	52%	64%	56%	48%	46%
	54-deoxyCTX1B	31%	20%	27%	35%	24%
	sum of 54-deoxyCTX1B and 52-epi-54deoxyCTX1B	83%	84%	83%	82%	71%
	ratio 54-deoxyCTX1B / 52-*epi*-54-deoxyCTX1B	1.7	3.3	2.1	1.4	1.9
5	CTX1B	17%	17%	19%	18%	31%
	52-*epi*-54-deoxyCTX1B	56%	56%	44%	46%	45%
	54-deoxyCTX1B	27%	27%	37%	37%	23%
	sum of 54-deoxyCTX1B and 52-epi-54deoxyCTX1B	83%	83%	81%	82%	69%
	ratio 54-deoxyCTX1B / 52-*epi*-54-deoxyCTX1B	2.1	2.0	1.2	1.3	1.9

Data refer to results obtained for the CTX1B calibrant, data provided as mean (n=3).

Table S12. Contents of CTX3C group analogues determined in sample 6 by each participant.

CTX analogue	Lab	calibrant			
		CTX1B (commercial)	CTX3C (commercial)	CTX1B (qNMR adjusted)	CTX3C (qNMR adjusted)
CTX3C	A	NA	NA	NA	NA
	B	0.155 ± 0.009	0.504 ± 0.031	0.083 ± 0.005	0.160 ± 0.010
	C	0.191 ± 0.011	0.442 ± 0.020	0.102 ± 0.006	0.140 ± 0.006
	D	NA	NA	NA	NA
	E	0.172 ± 0.008	0.175 ± 0.008	0.092 ± 0.004	0.055 ± 0.003
	all	0.173 ± 0.017	0.374 ± 0.153	0.093 ± 0.009	0.119 ± 0.049
49-*epi*-CTX3C	A	NA	NA	NA	NA
	B	0.667 ± 0.017	2.299 ± 0.060	0.359 ± 0.009	0.730 ± 0.019
	C	0.849 ± 0.050	1.691 ± 0.096	0.456 ± 0.027	0.537 ± 0.030
	D	0.486 ± 0.064	1.448 ± 0.202	0.261 ± 0.034	0.460 ± 0.064
	E	2.892 ± 0.139	2.921 ± 0.140	1.554 ± 0.074	0.928 ± 0.044
	all	1.224 ± 1.017	2.090 ± 0.608	0.657 ± 0.546	0.664 ± 0.193
51-hydroxyCTX3C	A	NA	NA	NA	NA
	B	0.487 ± 0.026	1.668 ± 0.091	0.262 ± 0.014	0.530 ± 0.029
	C	0.586 ± 0.049	1.193 ± 0.093	0.315 ± 0.026	0.379 ± 0.030
	D	0.103 ± 0.023	0.230 ± 0.075	0.056 ± 0.013	0.073 ± 0.024
	E	0.277 ± 0.007	0.281 ± 0.007	0.149 ± 0.004	0.089 ± 0.002
	all	0.364 ± 0.197	0.843 ± 0.641	0.195 ± 0.106	0.268 ± 0.204
2,3-dihydroxyCTX3C	A	NA	NA	NA	NA
	B	1.881 ± 0.140	6.550 ± 0.490	1.011 ± 0.075	2.081 ± 0.156
	C	2.253 ± 0.310	4.354 ± 0.589	1.210 ± 0.167	1.384 ± 0.187
	D	0.362 ± 0.033	1.054 ± 0.104	0.195 ± 0.018	0.335 ± 0.033
	E	5.666 ± 0.163	5.721 ± 0.165	3.044 ± 0.088	1.818 ± 0.052
	all	2.540 ± 2.031	4.420 ± 2.214	1.365 ± 1.091	1.404 ± 0.704
49-*epi*-2,3-dihydroxyCTX3C	A	NA	NA	NA	NA
	B	1.644 ± 0.139	5.718 ± 0.488	0.883 ± 0.075	1.817 ± 0.155
	C	1.286 ± 0.113	2.521 ± 0.215	0.691 ± 0.061	0.801 ± 0.068
	D	0.449 ± 0.036	1.329 ± 0.115	0.241 ± 0.019	0.422 ± 0.036
	E	7.299 ± 0.356	7.370 ± 0.360	3.921 ± 0.191	2.342 ± 0.114
	all	2.669 ± 2.833	4.234 ± 2.542	1.434 ± 1.522	1.345 ± 0.808
total content	A	NA	NA	NA	NA
	B	4.835 ± 0.309	16.739 ± 1.083	2.597 ± 0.166	5.319 ± 0.344
	C	5.165 ± 0.522	10.200 ± 0.991	2.775 ± 0.281	3.241 ± 0.315
	D	1.401 ± 0.048	4.061 ± 0.153	0.753 ± 0.026	1.290 ± 0.049
	E	16.305 ± 0.483	16.468 ± 0.487	8.760 ± 0.259	5.232 ± 0.155
	all	6.927 ± 5.871	11.867 ± 5.483	3.721 ± 3.154	3.771 ± 1.742

Contents provided in $\mu g\ kg^{-1}$ as mean ± SD for each lab (n=3) and for all labs combined (n=12 or n=9); three decimal places are provided for higher comprehensiveness of the data; ND – data not available as content was below the LOD of the system.

Table S13. Mean proportion of the analogues detected in samples 6.

	B	C	D	E
CTX3C	3%	4%	0%	1%
49-*epi*-CTX3C	14%	16%	35%	18%
51-hydroxyCTX3C	10%	11%	7%	2%
2,3-dihydroxyCTX3C	39%	44%	26%	35%
2,3-dihydroxy-49-*epi*-CTX3C	34%	25%	32%	45%
sum of CTX3C and 49-*epi*-CTX3C	17%	20%	35%	19%
sum of 2,3-dihydroxyCTX3C and 2,3-dihydroxy-49-*epi*-CTX3C	73%	68%	58%	80%

Data refer to results obtained for the CTX1B calibrant, data provided as mean (n=3).

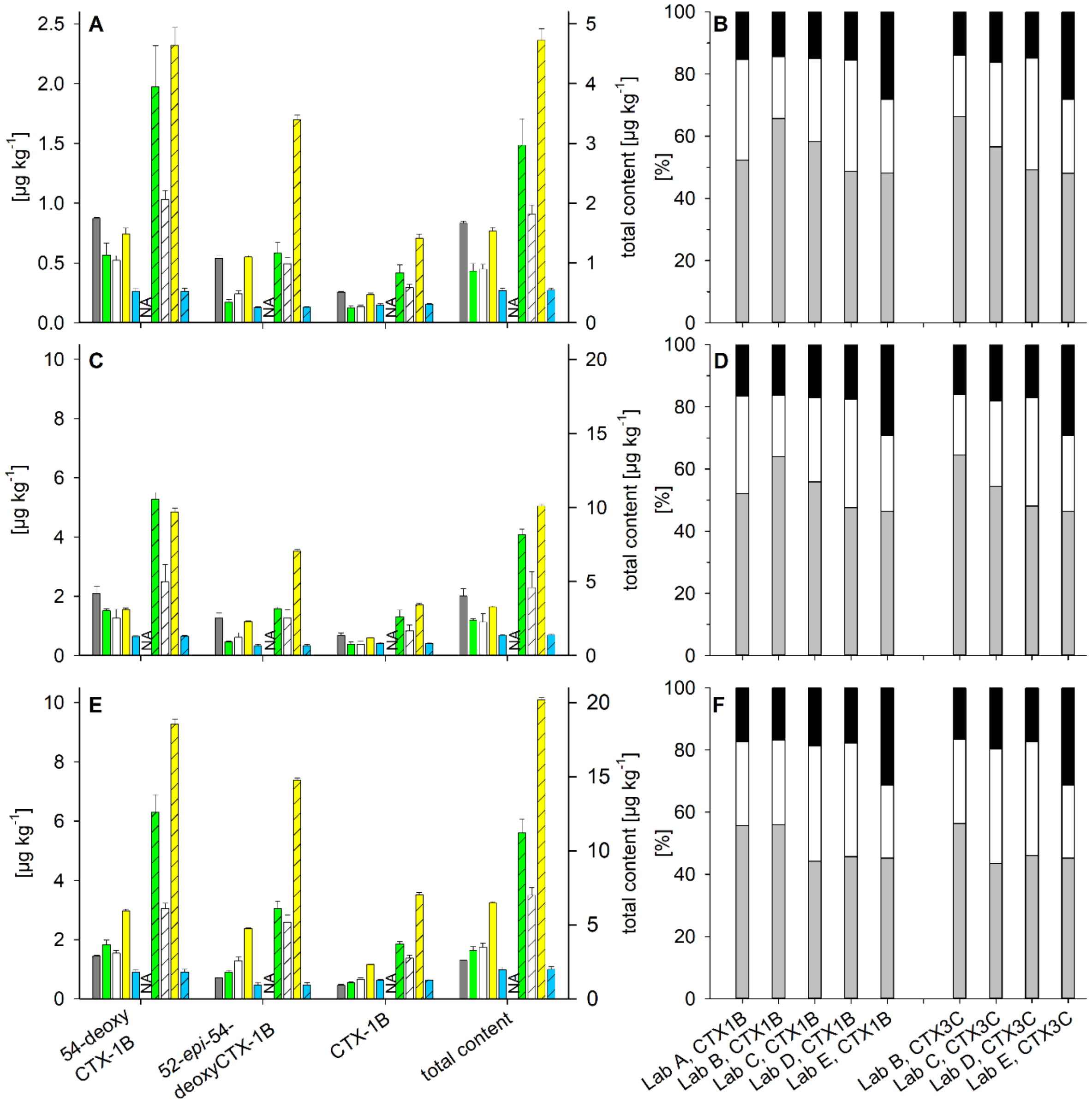

Figure S1. Quantitative (left, in color) and profile analysis results (right, gray scale) obtained for **(A,B)** sample 3 (fish curry, meal remnant), **(C,D)** sample 4 (fried fish fillet), and **(E,F)** sample 5 (fried fish fillet; see Table 1 for details); bars in the left panels represent the quantitative results obtained by (from left to right) Lab A (grey), B (green), C (white), D (yellow), and E (blue) via the calibration of CTX1B (blank) and CTX3C (shaded) **using the non-adjusted concentrations of the commercial standards**; results are provided as mean, error bar shows standard deviation (n=3); bars in the right panels represent profile analysis results obtained by each laboratory using the respective calibrant, segments represent portions of 54-deoxyCTX1B (grey), 52-*epi*-54-deoxyCTX1B (white) and CTX1B (black); NA – no data available for Lab A and the CTX3C calibration as the concentration of the standards was <LOD (11 µg L^{-1}); y-axes of panel C and E are adjusted to the same dimension to enable direct comparison of the results obtained for the same matrix (fried fish fillet) and different extraction methods (C: Spielmeyer et al. (2021), E: Murray et al. (2018)); second y-axes showing the total content are adjusted to twice the scale of the corresponding first y-axes.

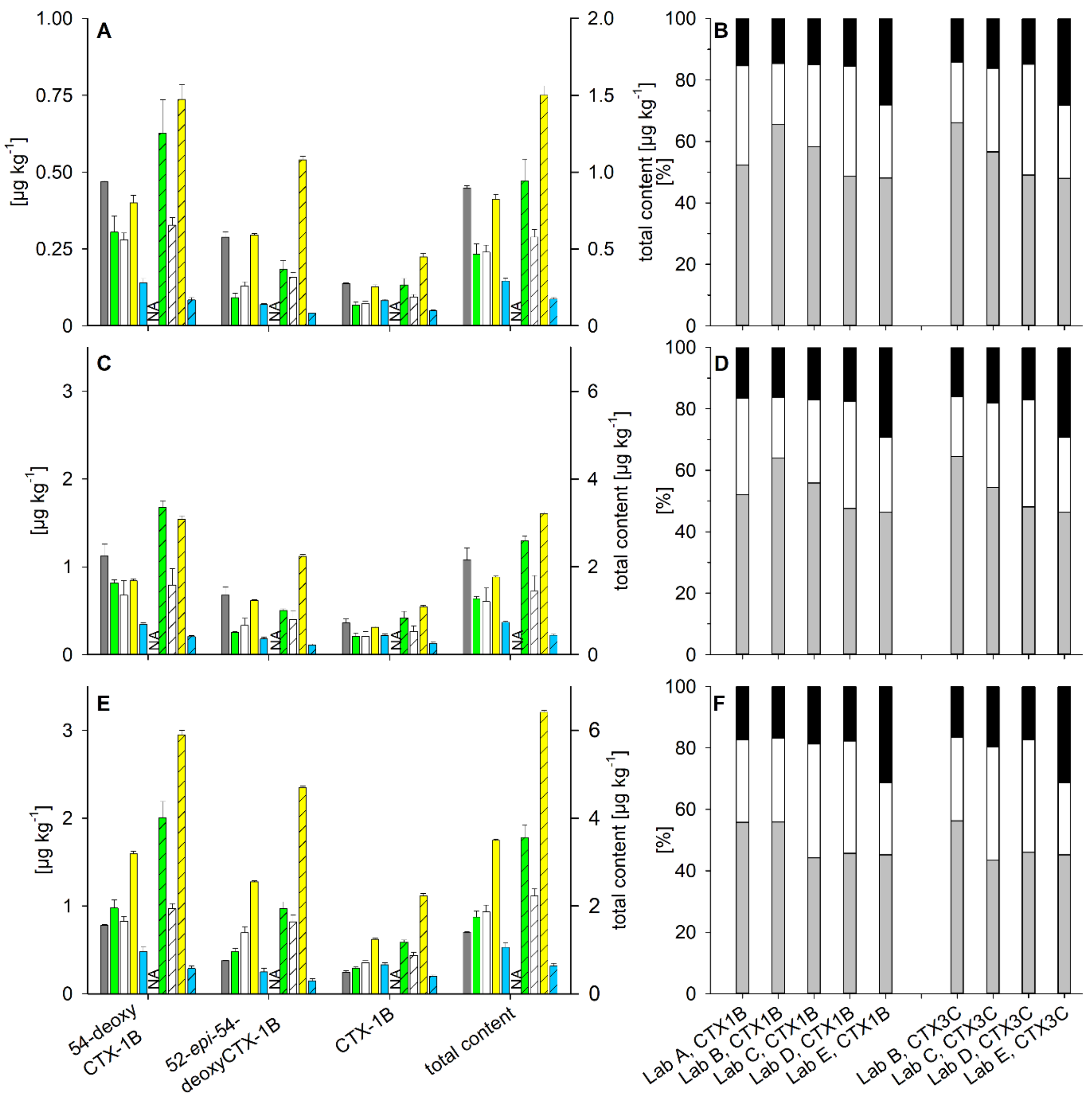

Figure S2. Quantitative (left, in color) and profile analysis results (right, gray scale) obtained for **(A,B)** sample 3 (fish curry, meal remnant), **(C,D)** sample 4 (fried fish fillet), and **(E,F)** sample 5 (fried fish fillet; see Table 1 for details); bars in the left panels represent the quantitative results obtained by (from left to right) Lab A (grey), B (green), C (white), D (yellow), and E (blue) via the calibration of CTX1B (blank) and CTX3C (shaded) **using the by qNMR adjusted concentrations of the commercial standards**; results are provided as mean, error bar shows standard deviation (n=3); bars in the right panels represent profile analysis results obtained by each laboratory using the respective calibrant, segments represent portions of 54-deoxyCTX1B (grey), 52-*epi*-54-deoxyCTX1B (white) and CTX1B (black); NA – no data available for Lab A and the CTX3C calibration as the concentration of the standards was <LOD (11 µg L^{-1}); y-axes of panel C and E are adjusted to the same dimension to enable direct comparison of the results obtained for the same matrix (fried fish fillet) and different extraction methods (C: Spielmeyer et al. (2021), E: Murray et al. (2018)); second y-axes showing the total content are adjusted to twice the scale of the corresponding first y-axes.

References

Kato, T., and Yasumoto, T. (2017). Quantification of Representative Ciguatoxins in the Pacific Using Quantitative Nuclear Magnetic Resonance Spectroscopy. *Marine Drugs* 15**,** 309, https://doi.org/10.3390/md15100309.

Murray, J.S., Boundy, M.J., Selwood, A.I., and Harwood, D.T. (2018). Development of an LC–MS/MS method to simultaneously monitor maitotoxins and selected ciguatoxins in algal cultures and P-CTX-1B in fish. *Harmful Algae* 80**,** 80-87, https://doi.org/10.1016/j.hal.2018.09.001.

Spielmeyer, A., Loeffler, C.R., and Bodi, D. (2021). Extraction and LC-MS/MS analysis of ciguatoxins: A semi-targeted approach designed for fish of unknown origin. *Toxins* 13**,** 630, https://doi.org/10.3390/toxins13090630.
